# Inactivation of *Notch4* Attenuated Pancreatic Tumorigenesis in Mice

**DOI:** 10.1158/2767-9764.CRC-22-0106

**Published:** 2022-12-12

**Authors:** Kiyoshi Saeki, Wanglong Qiu, Richard A. Friedman, Samuel Pan, Jordan Lu, Shu Ichimiya, Iok In Christine Chio, Carrie J. Shawber, Jan Kitajewski, Jianhua Hu, Gloria H. Su

**Affiliations:** 1 Herbert Irving Comprehensive Cancer Center, Columbia University Irving Medical Center, New York, New York.; 2 Department of Pathology & Cell Biology, Columbia University Irving Medical Center, New York, New York.; 3 Department of Biomedical Informatics, Columbia University Irving Medical Center, New York, New York.; 4 Department of Biostatistics, Columbia University Irving Medical Center, New York, New York.; 5 Institute for Cancer Genetics, Columbia University Irving Medical Center, New York, New York.; 6 Deparments of Obstetrics and Gynecology and Surgery, Columbia University Irving Medical Center, New York, New York.; 7 Department of Physiology and Biophysics, University of Illinois Cancer Center, University of Illinois Chicago, Chicago, Illinois.

## Abstract

Expression of the Notch family of receptors is often upregulated in pancreatic ductal adenocarcinoma (PDAC). In this study, we focused on Notch4, which had not been investigated in PDAC.

We generated KC (*LSL-Kras^G12D^;p48-Cre*), N4*^−^^/^^−^*KC (*Notch4^−^^/^^−^;LSL-Kras^G12D^;p48-Cre*), PKC (*p16^fl/fl^;LSL-Kras^G12D^;p48-Cre*), and N4*^−^^/^^−^*PKC (*Notch4^−/^^−^; p16^fl/f^^l^;LSL-Kras^G12D^;p48-Cre*) genetically engineered mouse models (GEMM). We performed caerulein treatment in both KC and N4*^−^^/^^−^*KC mice, and the development of acinar-to-ductal metaplasia (ADM) and pancreatic intraepithelial neoplasia (PanIN) lesions were significantly diminished in the N4*^−^^/^^−^*KC than in the KC GEMM (*P* = 0.01). This *in vivo* result was validated by *in vitro* ADM induction of the explant cultures of pancreatic acinar cells from the N4*^−^^/^^−^*KC and KC mice (*P* < 0.001), confirming that Notch4 is an important contributor to early pancreatic tumorigenesis.

To evaluate the role of Notch4 in the later stage of pancreatic tumorigenesis, we compared the PKC and N4*^−^^/^^−^*PKC mice. The N4*^−^^/^^−^*PKC mice had better overall survival (*P* = 0.012) and significantly reduced tumor burden (PanIN: *P* = 0.018 at 2 months, PDAC: *P* = 0.039 at 5 months) compared with the PKC GEMM. RNA-sequencing analysis of pancreatic tumor cell lines derived from the PKC and N4*^−^^/^^−^*PKC GEMMs revealed that 408 genes were differentially expressed (FDR < 0.05) and *Pcsk5* is a potential downstream effector of the Notch4 signaling pathway (*P* < 0.001). Low expression of Pcsk5 positively correlates with good survival in patients with PDAC (*P* = 0.028).

We have identified a novel role for Notch4 signaling with tumor-promoting function in pancreatic tumorigenesis. Our study also uncovered a novel association between *Pcsk5* and Notch4 signaling in PDAC.

**Significance:**

We demonstrated that global inactivation of *Notch4* significantly improved the survival of an aggressive mouse model for PDAC and provided preclinical evidence that Notch4 and Pcsk5 are novel targets for PDAC therapies.

## Introduction

Pancreatic ductal adenocarcinoma (PDAC) has one of the highest mortality rates among all types of cancers. For all stages combined, the 5-year survival rate is 9% (1). The development of early detection methods and effective therapies are needed to improve the outcome of patients with PDAC (2). Studies exploring the mechanisms and mutations associated with PDAC onset have established a major role played by activating mutations of *Kras* during the formation of acinar-to-ductal metaplasia (ADM) and pancreatic intraepithelial neoplasia (PanIN), which precede PDAC development (3, 4). In addition to *Kras* mutation, many signaling pathways involved in cell fate determination, such as Notch, Hedgehog, and the Wnt signaling, are known to be activated (5–9), but the precise roles of these pathways in pancreatic tumorigenesis warrant further elucidations.

Activation of the Notch signaling pathway requires cell–cell contact by Notch receptor–ligand interaction. The receptor–ligand interaction results in cleavage events by extracellular ADAM metalloproteases and an intracellular γ-secretase–containing complex, thereby releasing the intracellular domain of Notch (NICD). Once released, the NICD translocates into the nucleus where it interacts with the transcription factor RBP-J, building up a trimeric coactivator complex composed of RBP-J, MAML1 (mastermind-like 1), and NICD itself, together with additional coactivators (9, 10). All four vertebrate *Notch* genes (*Notch 1–4*) are expressed in the pancreas (11). In adult pancreatic tissues, the Notch signaling pathways prevent cells from terminally differentiation, and maintain the pools of undifferentiated stem and progenitor cells (12, 13). Notch signaling has been implicated in the pathogenesis of a number of malignancies, including pancreatic cancer (7, 14–16).

The putative oncogenic role of the Notch signaling pathway in pancreatic tumorigenesis has been investigated previously using genetically engineered mouse models (GEMM; refs. 17–20). Intriguingly, contradictory conclusions were drawn for the potential role of *Notch1* in pancreatic tumorigenesis based on these GEMM studies. De La and colleagues reported that Notch1 promoted oncogenic *Kras*-induced ADM/PanIN formation *in vivo* and hence concluded that *Notch1* serves as an oncogene in the context of *Notch1* and *Kras* coactivation (17). On the contrary, Hanlon and colleagues demonstrated that *Notch1* functions as a tumor suppressor gene because the loss of *Notch1* in the context of activated *Kras* led to increased PanIN incidence and progression (18). Thus, the role of Notch signaling in pancreatic cancer remains to be further elucidated. In addition, studies to date pertaining to the Notch signaling pathway have focused on *Notch1* (17, 18, 21) and *Notch2* (19, 21). In contrast, the role of *Notch4* in pancreatic cancer is largely unknown. Therefore, in this study, we investigated the contributions of *Notch1* and *Notch4* to the development of pancreatic cancer *in vivo*. We performed independent evaluations of the impacts of *Notch1* or *Notch4* loss in the context of previously established GEMMs for pancreatic cancer, *LSL-Kras^G12D^;p48-Cre* and *p16^fl/fl^;LSL-Kras^G12D^;p48-Cre* GEMMs (5, 22).

## Materials and Methods

### Mouse Strains

*LSL-Kras^G12D^;p48-Cre* (KC) and *p16^fl/fl^;LSL-Kras^G12D^;p48-Cre* (PKC) mice have been described in details previously (5, 22). Conventional heterozygous Notch1 deficient (*N1^+/^^−^*), conditional homozygous Notch1 deficient (*N1^fl/fl^*), and conventional homozygous Notch4 deficient (*N4^−^^/^^−^*) mice (23–25) were bred to KC and/or PKC GEMM to generate *Notch1^+/^^−^;p16^fl/fl^;LSL-Kras^G12D^;p48-Cre* (N1*^+/^^−^* PKC), *Notch1^fl/fl^;p16^fl/fl^;LSL-Kras^G12D^;p48-Cre* (N1 ^fl/fl^PKC), *Notch4^−^^/^^−^;LSL-Kras^G12D^;p48-Cre* (N4*^−^^/^^−^*KC), and *Notch4^−^^/^^−^; p16^fl/ fl^;LSL-Kras^G12D^;p48-Cre* (N4*^−^^/^^−^*PKC) GEMMs for this study. No developmental effects were observed in all of these mice. All mice were housed in the Animal Care Facility at Columbia University Irving Medical Center (CUIMC), and the studies were conducted in compliance with the CUIMC Institutional Animal Care and Use Committee guidelines.

### Histologic and Immunolabeling Analyses

Murine tissues were fixed in 10% neutral buffered formalin overnight, and embedded in paraffin. Routine hematoxylin and eosin (H&E) staining was performed by the Histology Service Core Facility at CUIMC.

For histologic analyses, each slide was examined in its entirety and the percentages of normal pancreas, ADM, PanIN, and PDAC areas in each mouse were calculated by their proportions to the entire mouse pancreas. When scoring the PanIN lesions alone, PanIN lesions were classified into three grades based on their architectural and cytologic atypia (PanIN-1, PanIN-2, and PanIN-3) first. Then the percentages of PanIN-1, PanIN-2, and PanIN-3 lesions in each mouse were calculated by the proportion of their areas to the total area of PanIN lesions in that mouse.

Unstained 5-mm sections derived from the formalin-fixed and paraffin-embedded blocks were deparaffinized in xylene three times and rehydrated in ethanol four times. Heat-induced antigen retrieval was performed on all slides in Tris-ethylenediaminetetraacetic acid (EDTA) buffer (0.5% Tween-20, citrate buffer, pH 6.8) in a steamer for 30 minutes. Slides were incubated with Dako peroxidase block buffer to block endogenous peroxidase activity. Primary antibody staining was performed at 4°C overnight. For IHC, the secondary antibodies were followed by a 40-minute incubation with universal secondary antibodies (Dako) and streptavidin–horseradish peroxidase. Hematoxylin was then used as counterstaining. Slides were dehydrated in ethanol and xylene and mounted with VectaMount permanent mounting medium (Vector Laboratories). For immunofluorescent (IF) double-staining assay, the fluorophore-conjugated secondary antibodies (Thermo Fisher Scientific) were incubated at room temperature for 2 hours. The Sirius red staining was performed as described previously (26). All other procedures were done according to the manufacture's instruction.

The primary antibodies used for IHC were Notch4 (1:1,000, Notch4-ICD, intracellular domain of Notch4), kindly provided by Dr. Carrie J. Shawber; refs. 27, 28), Amylase (1:100, catalog no.: sc-46657, Santa Cruz Biotechnology), CK19 (1:500, catalog no.: ab52625, Abcam), Pcsk5 (1:5,000, catalog no.: PA5-42378, Thermo Fisher Scientific), and endomucin (1:1,000, sc-65495, Santa Cruz Biotechnology). The primary antibodies used for IF were Notch4 (1:100, Notch4-ICD, kindly provided by Dr. Carrie J. Shawber; ref. 29), Amylase (1:100, catalog no.: sc-46657, Santa Cruz Biotechnology), CK19 (1:25, TROMA3; DSHB).

For CD68 IHC, antigen retrieval was done in 10 mmol/L citrate buffer (pH 6). Sections stained for CD68 were blocked for 1 hour in normal horse serum, 2.5%. CD68 (Abcam, catalog no. ab125212, RRID: AB_10975465, 1:500) was the primary antibody used.

### Caerulein Treatment

Acute pancreatitis was induced at the 6 weeks of age in KC and N4*^−^^/^^−^*KC mice by two sets of 6 hourly intraperitoneal caerulein injection (50 μg/kg diluted in saline; Sigma-Aldrich) separated by 24 hours. Control mice were injected with saline instead of caerulein. In this experiment, the final day of the caerulein/saline injection was considered day 0 (3, 30).

### Preparation of Epithelial Explant Cultures and Adenoviral Infection

Isolation of primary pancreatic acinar cells was modified from the previously published protocols (31, 32). Whole pancreas was harvested and digested in 0.2 mg/mL collagenase-P (Millipore Sigma) at 37°C. Following multiple washes with Hanks balanced salt solution supplemented with 5% FBS, collagenase-digested pancreatic tissue was sequentially filtered through 500 μm (Spectrum Laboratories) and 100 μm polypropylene mesh (VWR). The filtrate was passed through a 30% FBS cushion at 1,000 rpm. The cellular pellet was resuspended in Waymouths complete medium [Waymouths MB 752/1 media (US Biological) supplemented with penicillin G (1,000 U/mL), streptomycin (100 μg/mL), 0.1 mg/mL soybean trypsin inhibitor (Invitrogen-Thermo Fisher Scientific), 1 μg/mL dexamethasone (Sigma-Aldrich), and 2.5% heat-inactivated FBS]. Equal multiplicity of infection of AdCMVempty and Ad5CMVCre (Viral vector Core, The University of Iowa, Iowa City, IA) was added to the cellular pellet suspended medium and incubated at 37°C and 5% CO_2_ incubator for 4–6 hours in a 6-well plate. The floating acinar cells were collected and centrifuged. The supernatants were aspirated and the cellular pellets were resuspended with Waymouths complete medium. An equal volume of neutralized rat tail collagen type I (RTC; Corning) was added to the cellular suspension. Cellular/RTC suspension (500 μL) was pipetted into each well of a 24-well plate precoated with 200 μL of RTC. After solidification of the RTC, Waymouths complete medium were added. Cultures were maintained at 37°C and 5% CO_2_ in air for up to 7 days. For ADM quantification, ductal structure cells were quantified in each well and statistically evaluated.

### Single-cell RNA-sequencing Analysis

We used existing single-cell RNA-sequencing (scRNA-seq) data from Schlesinger and colleagues, who had performed scRNA-seq analyses of pancreatic tissues taken from *Ptf1a-CreER;LSL-KrasG12D;LSL-tdTomatao* mice at different timepoints after tamoxifen injection [Gene Expression Omnibus (GEO) Series accession number GSE141017] (33). In a similar manner to the original study (33), we used Seurat to identify major cell types using dimension reduction followed by clustering of cell groups. We then performed graph-based unsupervised clustering, uniform manifold approximation, and t-stochastic neighbor embedding, for data visualization in two-dimension space. After cell type identification (ductal, acinar, tumor, endocrine, fibroblast, immune, pericytes, and endothelial cells), the expressions of *Notch 1–4* for each cell type were annotated through dimensional reduction plots and violin plots. We removed cell lines from mice 6 weeks or below, and subsetted the data to only the cell types acinar, tumor, and ductal, before producing more dimensional reduction plots and violin plots for *Notch 1–4*.

### Molecular Analyses

Mouse pancreatic tumors were resected and cultured to establish primary pancreatic tumor cell lines as described previously (34). Total RNAs of primary pancreatic tumor cell lines were extracted for subsequent PCR, RT-PCR, qRT-PCR, and sequencing as described in the same publication (22, 34, 35). RNA-seq analyses were performed on PKC (*n* = 3) and N4*^−^^/^^−^*PKC (*n* = 2) tumor cell lines at the Columbia Genome Center.

#### qRT-PCR

Total RNA of PKC and N4^−/−^PKC pancreatic tumor cell lines were extracted using the miRNeasy Mini kit (Qiagen), quantified by spectrophotometry (Ultraspec2100; Amersham Pharmacia Biotech), and reversed transcribed with SuperScriptⅢ Reverse Transcriptase (Invitrogen) according to the manufacturer's protocol.

For the real-time RT-PCR, 1 μg of RNA was treated with DNase and reverse transcribed to cDNA with a Quantitect Reverse Transcription Kit (Qiagen) according to the manufacturer's protocol. Reactions were run with POWER SYBR Green PCR Master Mix (Thermo Fisher Scientific) on a StepOnePlus Real-Time PCR System (Applied Biosystems). Each sample was tested in triplicate to confirm the reproducibility of the results. All the experiments were done in triplicate and repeated three times on different days.

The primer sequences used were as follows: for *Pcsk5*, 5′-TGTCTGTGGGAAATGCAGTGA-3′ and 5′-AACCCGCCCTTGCACTCT-3′; for *NGF*, 5′-AGGCCCATGGTACAATCCCTTTCA-3′ and 5′-ATCTCCAACCCACACACTGACACT-3′; for *β-actin*, 5′-GGCTGTATTCCCCTCCATCG-3′ and 5′-CCAGTTGGTAACAATGCCATGT-3′. The amount of each target gene in a given sample was normalized to the level of β-actin.

#### RNA-seq Analyses

RNA was enriched by poly‐A pulldown. Library preparation was performed with the Illumina TruSeq RNA prep kit.

A total of 60M 100 bp paired end reads per sample were sequenced with the Illumina 2000. Reads were aligned to the mm9 build of the mouse genome with BowTie (36) and TopHat (37). Reads were quantified with featureCounts (38). Data were deposited in GEO GSE184060. Differential expression was analyzed using DeSeq (39), with a significance cutoff of the Benjamini–Hochberg FDR (40), FDR ≤ 0.05. The Volcano plot (41) was generated from differential expression results using R base graphics (42). Statistically significantly differentially expressed genes were compared with the Reactome Pathway database (43) using overrepresentation analysis, implemented in WEBGESTALT (44), to the Gene Ontology Biological Process database (45) using the Elim method (46), implemented in iPathwayGuide (47), and to the Kyoto Encyclopedia of Genes and Genomes (48) database using the signaling pathway impact analysis (49, 50) method as implemented in iPathwayGuide.

### Cell Proliferation Assay

All PDAC cell lines were seeded onto 96-well plates at 5,000 cells/well and they were treated with or without 100 μmol/L of CMK (decanoyl-Arg-Val-Lys-Arg-chloromethlketone), and then they were cultured for 24, 48, and 72 hours. Cell proliferation was assessed by absorbance (Varioskan LUX multimode microplate reader; Thermo Fisher Scientific) at 592 nm (reference wavelength: 620 nm) using Cell proliferation Kit (MTT; Roche Diagnostic GmbH).

Proprotein convertases (PC) inhibitor, CMK (decanoyl-Arg-Val-Lys-Arg-chloromethylketone), was purchased from Enzo Life Sciences. Stocks were prepared at 10 mmol/L in ultrapure distilled water and dilutions were made directly before use.

### Statistical Analysis

The statistical analysis of survival curves was performed with EZR (51), which is an R package. Quantifications for endomucin expression levels were carried out using Image J (NIH, Bethesda, MD). A log-rank test was used to evaluate statistical significance of group differences in survival based on the Kaplan–Meier survival curves. All experiments were performed at least three independent times, unless otherwise noted. Data were expressed as means ± SD. Paired two-tailed *t* tests were used to analyze the data, unless noted otherwise. *P* < 0.05 were determined to be significant. Asterisks denote *P* values as follows: *, *P* < 0.05; ** , *P* < 0.01; ***, *P* < 0.001.

### Bioinformatics Analysis

The publicly available overall survival dataset (containing the published data for 176 patients) were obtained from Human Protein Atlas (https://www.proteinatlas.org/ENSG00000099139-PCSK5/pathology/pancreatic+cancer).

### Data Availability Statement

The data generated in this study are publicly available in GEO at GSE141017.

### Cell Lines Authentication

Human pancreatic cancer cell lines were purchased from the ATCC for this project, authenticated by the company, and passaged less than 6 months. Primary mouse pancreatic cancer cell lines were derived from our mouse models, authenticated by PCR-based genotyping, and passaged less than 10 times on average. *Mycoplasma* testing was periodically performed but not routinely done on short-term cultures.

## Results

### Inactivation of *Notch4* Delayed the Development of PDAC and Led to Favorable Outcomes

**FIGURE 1:**
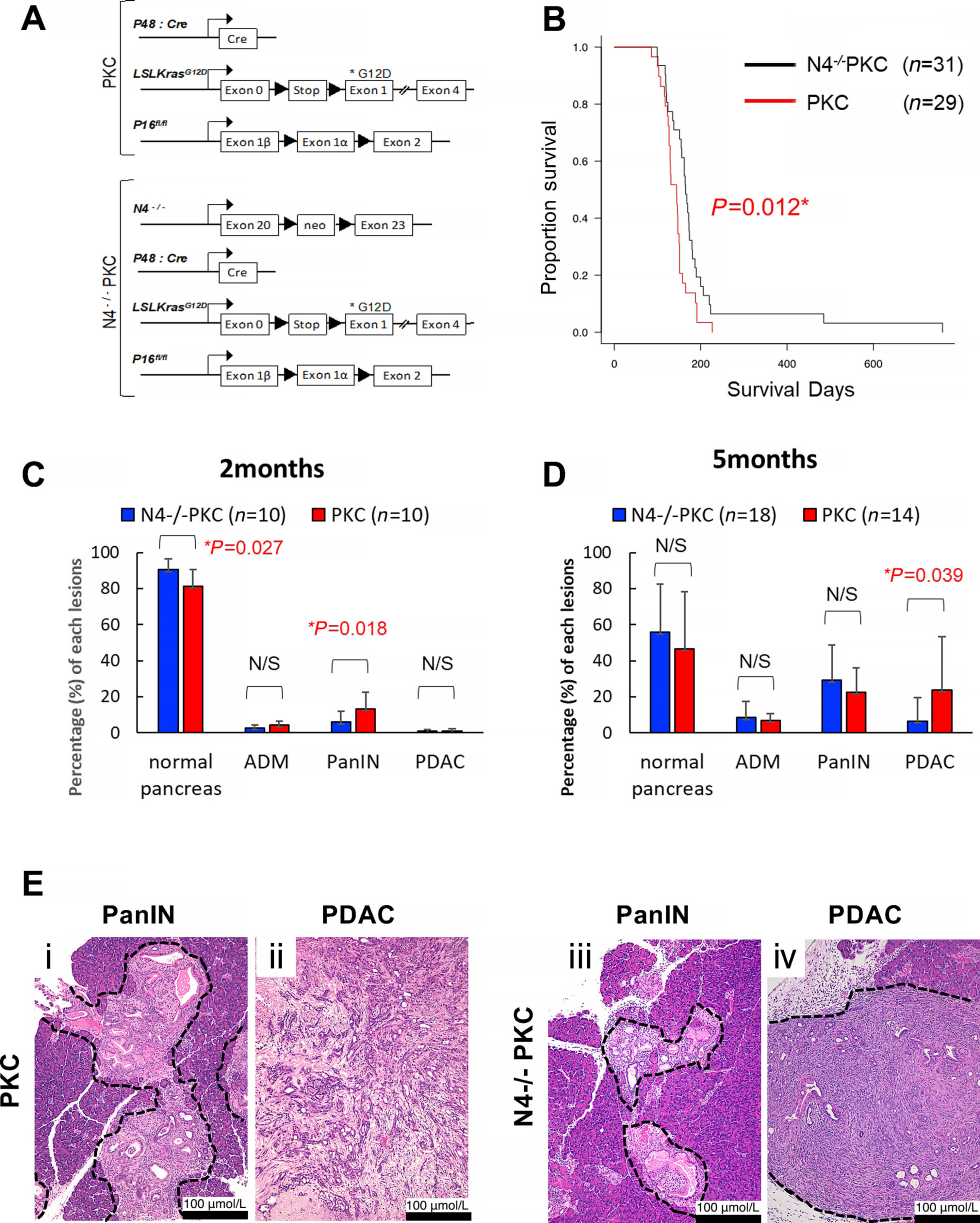
Loss of *Notch4* led to better prognosis in PDAC. **A,** Schematics of PKC mouse model of PDAC, which uses *p16^fl/fl^* (P), *LSL-KrasG^12D^* (K), and *p48-Cre* (C). Conventional homozygous deletion of *Notch4* in PDAC was attained by crossing the PKC with *Notch4^−^^/^^−^* mouse strain and is referred to as N4^−/−^PKC. **B,** Kaplan–Meier analysis comparing the overall survival of the N4^−/−^PKC (*n* = 31) and the PKC (*n* = 29) mice. *, *P* < 0.05 by log-rank test. **C,** Evaluation of comparative percentages of normal pancreas, ADM, PanIN, and PDAC areas between the N4^−/−^PKC and the PKC mice at the age of 2 months. Loss of *Notch4* led to the suppression of PanIN formation in the N4^−/−^PKC mice. *, *P* < 0.05 by *t* test. **D,** Similar evaluation at the age of 5 months revealed that loss of *Notch4* resulted in the reduction of PDAC formation in the N4^−/−^PKC mice. *, *P* < 0.05 by *t* test. **E,** Representative H&E from the PKC and the N4^−/−^PKC mice at 2 months of age (PanIN, i, iii) and 5 months of age (PDAC, ii, iv). Dots surrounded area indicates the PanIN and PDAC lesions of the pancreas. Folds of magnification are 100×.

To investigate the roles of *Notch1* and *Notch4* in PDAC *in vivo*, we used a previously published PDAC mouse model that harbors the *p16^fl/fl^;LSL-Kras^G12D^;p48-Cre* (PKC) alleles and spontaneously develops PDAC and metastasis at 100% frequency (Fig. 1A; ref. 22). Because the expression of Notch1 is not restricted to the ductal epithelial cells and *Notch1^−^^/^^−^* mice died *in utero* (24), to evaluate the impact of Notch1 inactivation in tumor-intrinsic and -extrinsic manners, we generated *Notch1^+/^^−^;p16^fl/fl^;LSL-Kras^G12D^;p48-Cre* (N1*^+/^^−^*PKC) and *Notch1^fl/fl^;p16^fl/fl^;LSL-Kras^G12D^;p48-Cre* (N1^fl/fl^PKC; Supplementary Fig. S1A). The survival analyses were performed between the N1*^+/^^−^*PKC (*n* = 20) and the PKC (*n* = 29; Supplementary Fig. S1B), and also between the N1^fl/fl^PKC (*n* = 54) and the PKC (*n* = 29; Supplementary Fig. S1C). Tumor burdens of the PKC, N1*^+/^^−^*PKC, and the N1^fl/fl^PKC were also evaluated at the timepoints of 2 and 5 months of age using histopathologic classifications such as normal pancreas, ADM, PanIN, and PDAC (Supplementary Figs. S2–S4). We observed no statistically significant differences in the prognosis between the N1*^+/^^−^*PKC and the PKC GEMMs (*P* = 0.84; Supplementary Fig. S1B), nor between the N1^fl/fl^PKC and the PKC groups (*P* = 0.71; Supplementary Fig. S1C). Consistent with the survival analyses, there were no statistically significant differences in the histologic comparisons between the N1*^+/^^−^*PKC and the PKC groups (Supplementary Figs. S2A, S2B, S4A, and S4B), nor between the N1 ^fl/fl^PKC and the PKC cohorts (Supplementary Figs. S2C, S2D, S4C, and S4D). These results differ from the previous findings on *Notch1*, which purported either an oncogenic or a tumor-suppressive role for *Notch1* (17, 18). In this study, we found neither biallelic inactivation of *Notch1* in the tumor cells nor heterozygous deletion of *Notch1* globally exerted a statistically significant impact on pancreatic tumorigenesis driven by oncogenic *Kras* in the context of *p16* inactivation.

In contrast to the findings on *Notch1*, inactivation of *Notch4* in the PKC GEMM (*Notch4^−^^/^^−^;p16^fl/fl^;LSL-Kras^G12D^;p48-Cre* or N4*^−^^/^^−^*PKC mice hereafter) resulted in significant favorable outcomes. The conventional *Notch4* knockout mouse line was selected because *Notch4*^−/−^ mice are live born and fertile (23), and *Notch4^f/f^* mouse line is not yet available. The survival analysis was performed between the N4*^−^^/^^−^*PKC (*n* = 31) and the PKC (*n* = 29) GEMMs, and the N4*^−^^/^^−^*PKC mice displayed a statistically significantly better survival than the PKC mice (*P* = 0.012; Fig. 1B). We evaluated the histopathology of the N4*^−^^/^^−^*PKC and the PKC mice at the timepoints of 2 and 5 months of age (Fig. 1C and D). At 2 months of age, when evaluating the whole pancreases, the proportion of normal pancreas area in the N4*^−^^/^^−^*PKC mice was statistically significantly higher than that of the PKC mice (*P* = 0.027) and the percentage of PanIN lesions in the N4*^−^^/^^−^*PKC mice was statistically significantly lower than that of the PKC mice (*P* = 0.018; Fig. 1C–E). When comparing only the PanIN lesions, the N4*^−^^/^^−^*PKC mice tended to display fewer PanIN-3 lesions than the PKC mice at both the ages of 2- and 5-month timepoints, albeit the differences did not reach statistical significance (*P* = 0.054 and *P* = 0.169, respectively; Supplementary Fig. S5A and S5B). At 5 months of age, the proportion of PDAC lesions in the N4*^−^^/^^−^*PKC mice was statistically significantly lower than that of the PKC mice (*P* = 0.039; Fig. 1D and E). These results unambiguously indicate that the inactivation of *Notch4* in the context of both activated *Kras* and deleted *p16* led to preferable outcomes.

Intriguingly, mutational analysis of The Cancer Genome Atlas (TCGA) using cBioPortal revealed that alterations at *NOTCH4* are associated with better median survival among patients with PDAC (37.15 vs. 19.96 months, 95% confidence intreval). However, this difference in overall survival lacks statistical significance (*P* = 0.465), probably because the mutation frequency of *NOTCH4* among patients with PDAC is low (3.26%) and the sample size is limited in TCGA.

### Notch4 Expression was Highly Upregulated in ADM/PanIN Lesions Detected by IHC and IF

**FIGURE 2:**
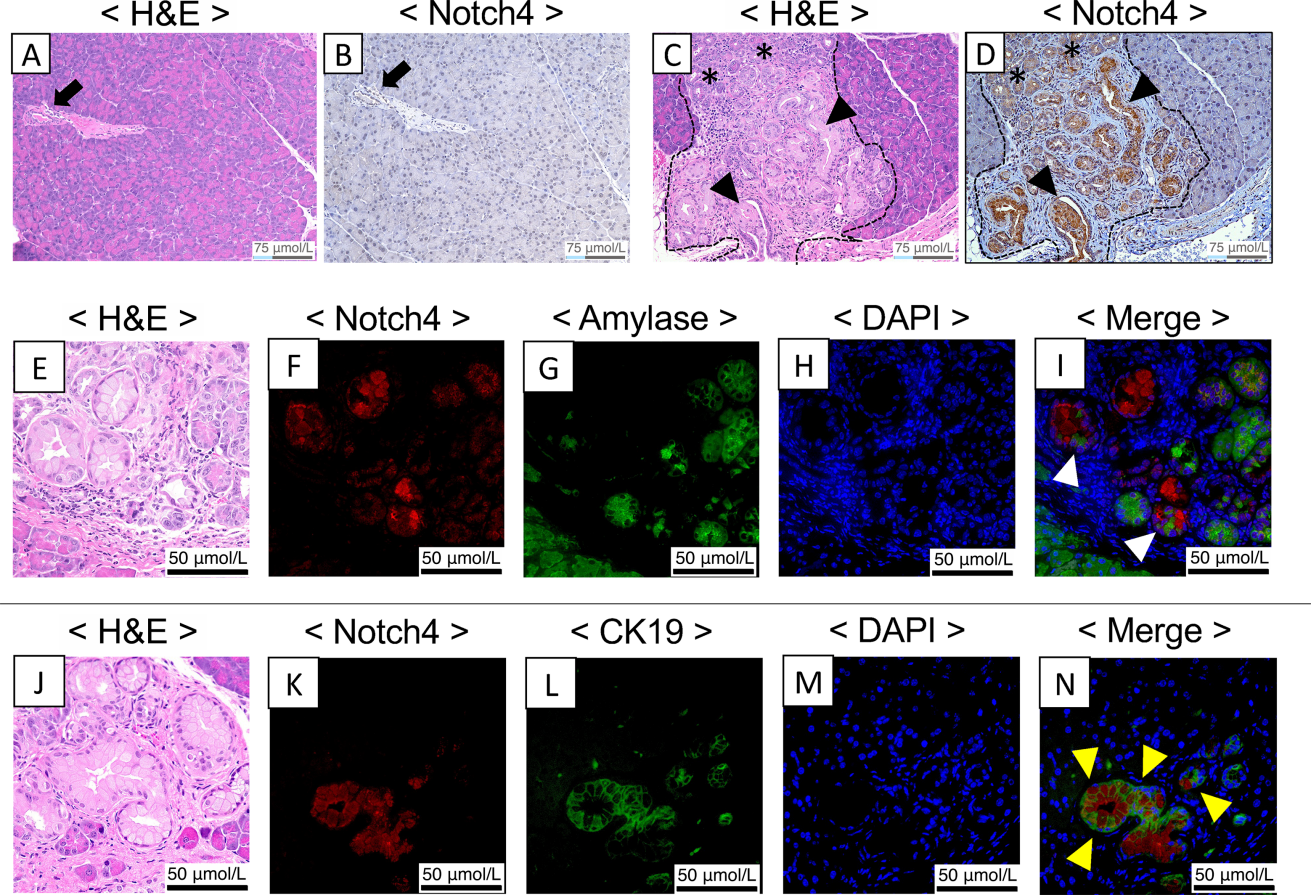
Upregulated expression of Notch4-ICD was detected in ADM/PanIN lesions by IHC and IF. Notch4-ICD expression was not detected in the normal pancreas area. Representative images of H&E staining (**A**) and Notch4-ICD immunolabeling by IHC (**B**) of the normal pancreas area of the PKC mouse at 2 months of age. Arrow points to normal ductal structure. **C** and **D,** High expression of Notch4-ICD was detected in the ADM/PanIN lesions by IHC. Representative images of H&E staining (**C**) and Notch4-ICD immunolabeling by IHC (**D**) of the ADM/PanIN lesions of the PKC mouse at 2 months of age. Dots surrounded area indicates the ADM/PanIN lesions of the pancreas. Stars point to ADM structures while arrowheads point to PanIN structures. **E–N,** High expression of activated Notch4-ICD in the ADM/PanIN lesions was confirmed by IF. Serial sections of the ADM/PanIN lesions of the PKC mouse (2 months of age) were stained by H&E (**E, J**), DAPI (**H, M**), or immunolabeled using antibodies against Notch4-ICD (**F, K**), amylase (**G**), or CK19 (**L**). Overexpression of Notch4-ICD (nuclear localization) was detected in some acinar (cytoplasmic amylase expression) and ductal (membranous CK19 expression) cells in the ADM/PanIN lesions (**I, N**). White arrowheads point to Notch4 and amylase colocalized area while yellow arrowheads point to Notch4 and CK19 colocalized area. Folds of magnification are 200× for **A**–**D** and 400× for **E**–**N**, respectively.

To understand the role of *Notch4* in pancreatic tumorigenesis further, we examined the expression levels of Notch4-ICD in the pancreatic tumors developed in the PKC GEMM by IHC and IF. Because pronounced histologic differences were detected in respect to the proportion of normal pancreas area (*P* = 0.027) and PanIN lesions (*P* = 0.018) between the N4*^−^^/^^−^*PKC and the PKC mice (Fig. 1C–E), and the inactivation of *Notch4* resulted in the reduction of PanIN lesions in the N4*^−^^/^^−^*PKC mice compared with the PKC mice (Fig. 1C and D), we focused on evaluating Notch4-ICD expressions in the ADM and the PanIN lesions. Highly upregulated Notch4-ICD expression was detected in the ADM and PanIN lesions compared with the normal tissues by IHC (Fig. 2A and D; Supplementary Fig. S6A–S6D). Elevated Notch4-ICD expression was also detected in the PDAC regions, but the expression pattern was heterogeneous, with a mixed of high and low areas, and slightly attenuated compared with the ADM and PanIN regions (Supplementary Fig. S6E–S6H). Using IF, we confirmed the overexpression of Notch4-ICD in the ADM and PanIN lesions of the PKC GEMM (Fig. 2E–N). The expression of Notch4-ICD also colocalized with some amylase or CK19^+^ cells (Fig. 2I and N). These results suggest that *Notch4* may contribute to ADM and PanIN formation *in vivo*.

### Inactivation of *Notch4* Attenuated the ADM/PanIN Progression Induced by Oncogenic *Kras* and Caerulein Treatment

To further test the hypothesis that the deletion of *Notch4* may affect pancreatic tumorigenesis in the early stage, we employed a caerulein-induced pancreatitis model. Acute chemical pancreatitis induced by the cholecystokinin receptor agonist caerulein treatment is known to accelerate the development of ADM/PanIN progression when mutant Kras is expressed in the acinar and/or centroacinar compartments (3, 30, 52). Caerulein treatment is therefore regarded as a useful method to evaluate the early stage of pancreas tumorigenesis.

**FIGURE 3:**
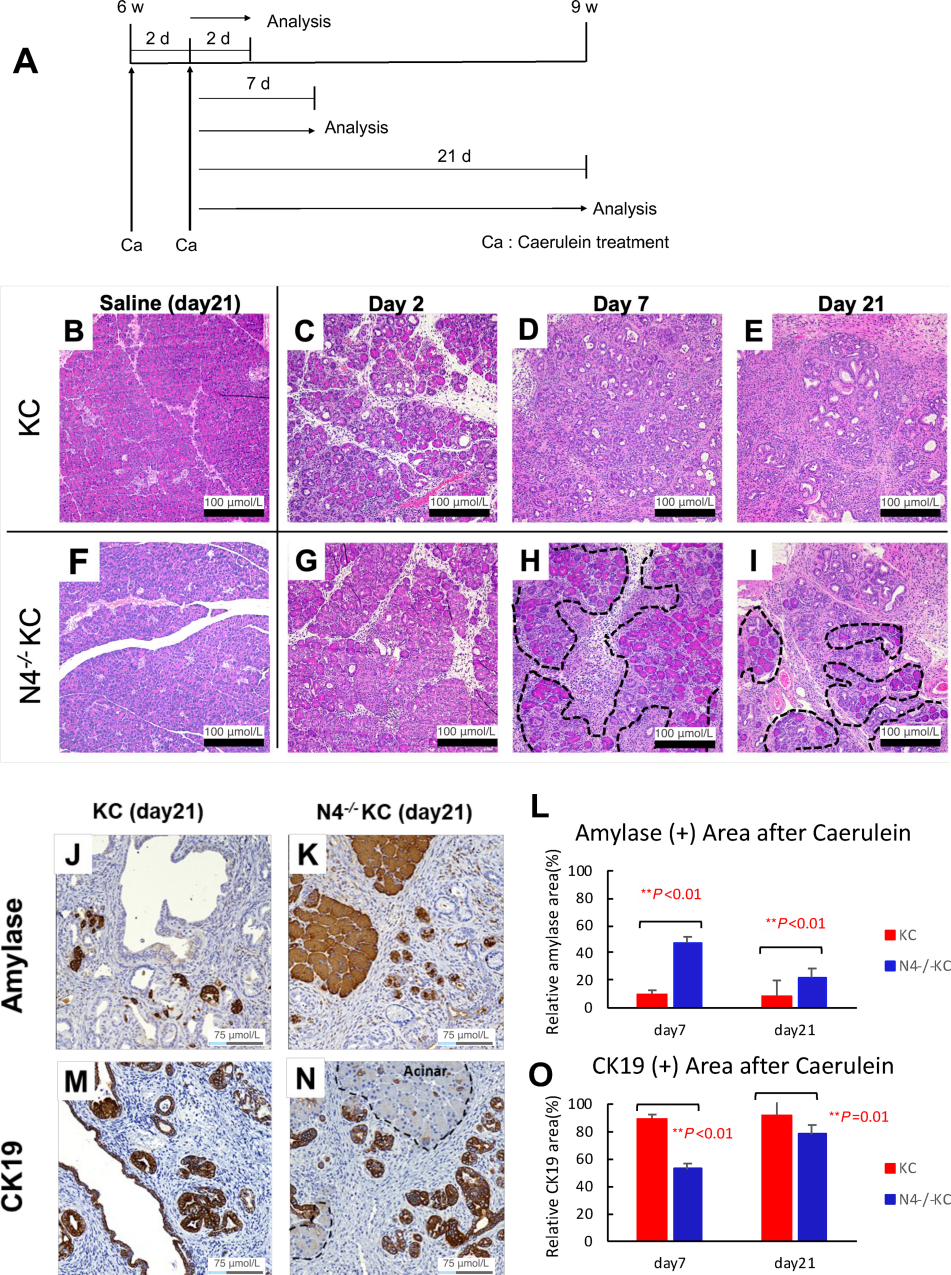
Inactivation of *Notch4* attenuated the ADM/PanIN progression induced by oncogenic Kras and Caerulein treatment. **A–I,** At 6 weeks of age, KC and N4^−/−^KC mice were treated with two rounds of caerulein (Ca) injections on alternative days or saline as the control, and analyzed 2, 7, or 21 days (d) later. Representative images of H&E staining of the pancreases of the KC and the N4^−/−^KC mice from the saline control group (**B, F**) or treated with the caerulein injection (**C–E, G–H**) at timepoints of 2, 7, and 21 days later. **B**–**I**; Folds of magnification are 100×. **J–O**, Immunolabeling of the pancreases of the KC and the N4^−/−^KC mice at the timepoint 21 days after the caerulein injection with antibodies to Amylase (**J, K**) or CK19 (**M, N**). Folds of magnification are 200×. **L,** Quantification of the Amylase-positive pancreatic areas revealed that the percentage of amylase-positive area in the N4*^−^^/^^−^*KC mice was significantly higher than that of the KC mice at 7 and 21 days after the caerulein treatment. **, *P* < 0.01 by *t* test. **O,** Quantification of the CK19-positive pancreatic areas revealed a significant reduction of PanIN lesions in the N4^−/−^KC mice compared with the KC mice at 7 and 21 days after the caerulein treatment. Dotted black lines in **H, I,** and **N** indicate the remaining acinar cell areas. **, *P* < 0.01 by *t* test.

We performed caerulein treatment on the KC and the N4*^−^^/^^−^*KC mice (Fig. 3), and then analyzed the ADM/PanIN lesions at timepoints of day 2, day 7, and day 21 (Fig. 3A). Saline-treated controls were also included for each genotype at each timepoint (Fig. 3B–F, and data not shown). At day 2, most of the pancreases in the caerulein-treated KC and N4*^−^^/^^−^*KC mice were comprised of acinar cells and only few lesions were replaced by duct cells (Fig. 3C–G). At day 7, while a large number of acinar cells were replaced by duct cells in the KC mice (Fig. 3D), acinar cells remained relatively intact in the N4*^−^^/^^−^*KC mice (Fig. 3H). By day 21, in the KC mice, almost the entire pancreas was replaced by ADM/PanIN lesions (Fig. 3E), but acinar cells still remained intact in the N4*^−^^/^^−^*KC mice (Fig. 3I). Saline-treated controls remained unaffected throughout the time course (Fig. 3B–F, and data not shown). To quantify the number of acinar cells, we performed amylase IHC on the pancreases of the caerulein-treated KC and the N4*^−^^/^^−^*KC mice and measured the amylase positive areas (Fig. 3J–L). At day 7 and day 21, the percentage of amylase-positive area in the N4*^−^^/^^−^*KC mice was significantly higher than that of the KC mice (*P* < 0.01; day 7 and day 21; Fig. 3J–L). We also performed CK19 IHC analysis in each of the KC and N4*^−^^/^^−^*KC mice at day 7 and day 21 and quantified the CK19-positive areas, which consisted mostly of neoplastic ductal epithelial cells that had undergone ADM and PanIN transformation (Fig. 3M–O). At day 7 and day 21, the proportion of CK19-positive area in the N4*^−^^/^^−^*KC mice was significantly lower than that of the KC mice (*P* < 0.01; day 7, *P* = 0.01; day 21; Fig. 3M–O). These results suggest that Notch4 is critical for the ADM and PanIN formation *in vivo* induced by pancreatic injury in the presence of mutant Kras^G12D^.

### Notch4 Expression Affected ADM Formation *In Vitro* Using the Explant Acinar Cell Culture

**FIGURE 4:**
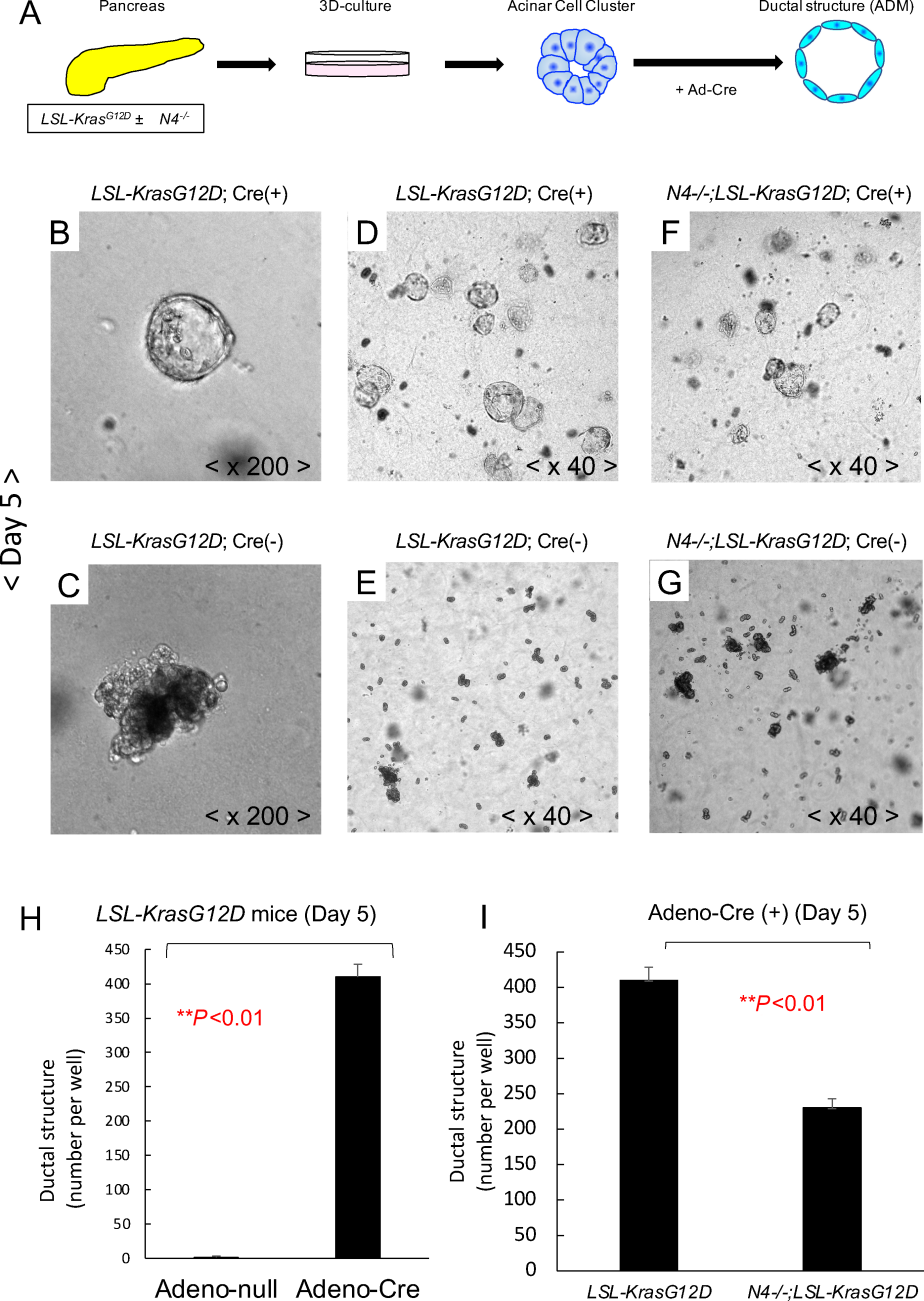
Notch4 plays an important role in the ADM process. **A,** A schematic showing the *in vitro* assay for Kras^G12D^-induced ADM. Cells were isolated from *LSL-Kras^G12D^* or *Notch4^−^^/^^−^;LSL-Kras^G12D^* mice and infected with Adeno-null or Adeno-Cre on day 0. The pancreatic acinar cell clusters from *LSL-Kras^G12D^* mice with Adeno-Cre led to the induction of ADM formation in sharp contrast to Adeno-null (representative brightfield images, **B**–**E**; folds of magnification are 40× and 200×, respectively). **H,** Quantification of the ductal structures developed in the acinar explant cultures with Adeno-null or Adeno-Cre. **, *P* < 0.01 by *t* test. The pancreatic acinar cell clusters from *Notch4^−^^/^^−^;LSL-Kras^G12D^* mice with Adeno-Cre led to reduced induction of ADM formation when compared with those from *LSL-Kras^G12D^* mice (representative brightfield images, **D** and **F**; folds magnification are 40×). Adeno-null treated *LSL-Kras^G12D^* or *N4^−^^/^^−^;LSL-Kras^G12D^* explant cultures served as the negative control (**E** and **G**; magnification is 40-folds). **I,** Quantification of ductal structures of the *LSL-Kras^G12D^* or the *N4^−/−^;LSL-Kras^G12D^* explant cultures with Adeno-Cre. **, *P* < 0.01 by *t* test.

To determine the role of Notch4 on the ADM process further, we used the established *in vitro* explant acinar cell three-dimensional (3D) culture (31, 32) in which mouse primary pancreatic acinar cells are isolated and then reseeded *ex vivo* in 3D cell culture to induce ADM (Fig. 4). The explant cultures of pancreas which developed progressive conversion from an acinar cell-predominant phenotype to a ductal structure phenotype were regarded as ADM events.

We isolated acinar cells from both *LSL-Kras^G12D^* mice and *Notch4^−^^/^^−^;LSL-Kras^G12D^* mice, and induced the expression of the *Kras^G12D^* allele with the Adeno-null or Adeno-Cre (Fig. 4A). Then, we evaluated the number of ADM events between *LSL-Kras^G12D^* with or without Adeno-Cre (Fig. 4B–H), and between the *LSL-Kras^G12D^* and *Notch4^−^^/^^−^;LSL-Kras^G12D^* genotypes (Fig. 4D–I). As expected, expression of oncogenic Kras induced by Adeno-Cre in acinar cells led to a dramatic increase in ADM events at the timepoint of day 5 (Fig. 4B–H). The number of ADM events in the *Notch4^−^^/^^−^;LSL-Kras^G12D^* with Adeno-Cre group was significantly lower than the *LSL-Kras^G12D^* with Adeno-Cre group (*P* < 0.01; Fig. 4D–I). These results confirm that Notch4 is an important contributor to Kras-mediated acinar to ductal metaplasia.

### *Notch4* Deficiency was Associated with Decreased Tumor Angiogenesis and Desmoplastic Reaction

**FIGURE 5:**
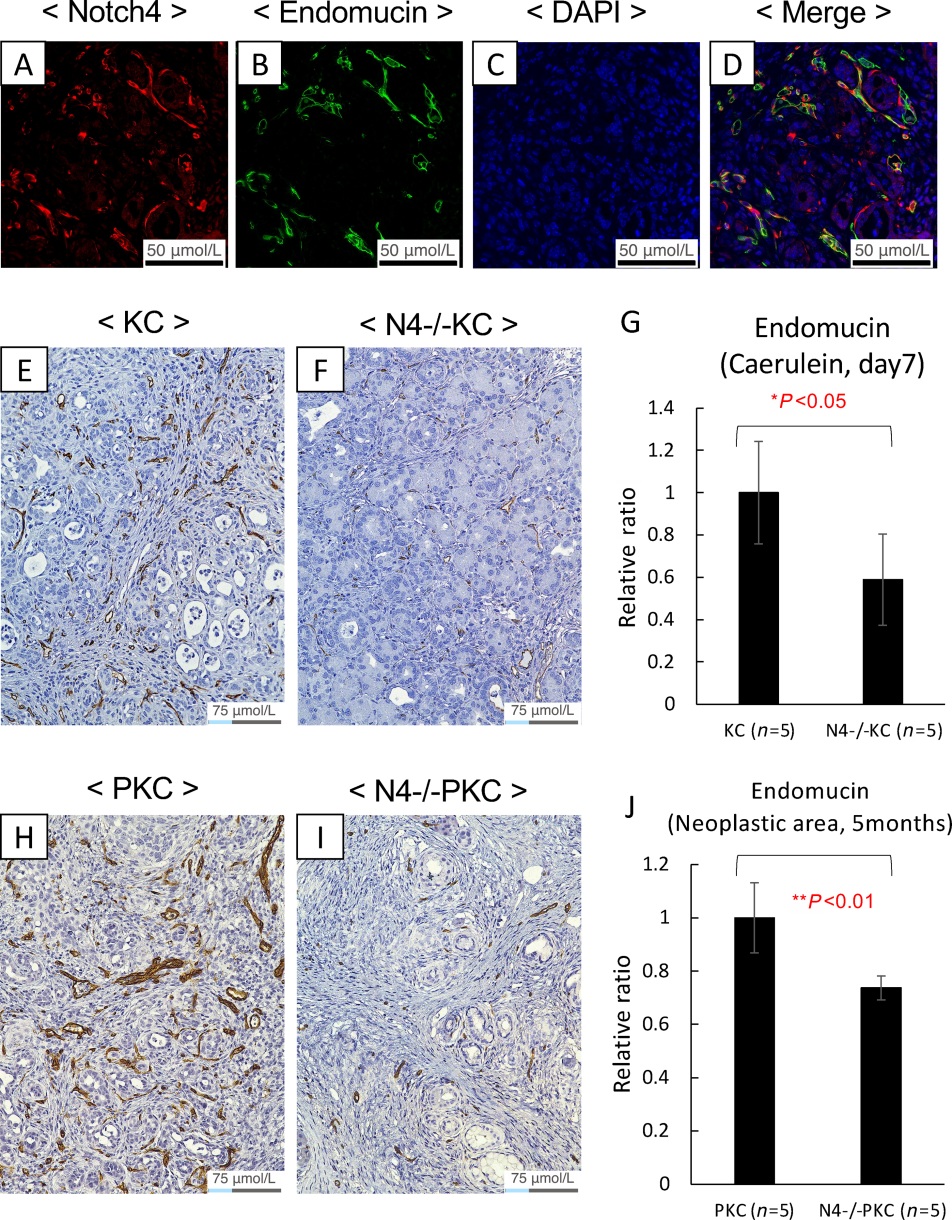
*Notch4* loss in the endothelial compartment was associated with reduced tumor burden. **A–D**, High expression of Notch4-ICD was detected in the endothelial cells in the pancreases of the KC mice treated with Caerulein injection. Pancreatic tissues from timepoint 7 days posttreatment were immunolabeled with antibodies against Notch4-ICD (**A**) or endomucin (**B**), and counterstained with DAPI (**C**). Notch4-ICD and endomucin were seen colocalized by IF. **E** and **F,** Representative images of endomucin immunolabeling of the pancreases of the KC and the N4^−/−^KC mice at the timepoint 7 days after caerulein injection. **G,** Quantification of the endomucin-positive pancreatic areas revealed that the percentage of endomucin-positive area in the KC mice was significantly higher than that of the N4*^−^^/^^−^*KC mice at 7 days after the caerulein treatment. *, *P* < 0.05 by *t* test. **H** and **I,** Representative images of endomucin immunolabeling of the pancreases of the PKC and the N4^−/−^PKC mice at the timepoint of 5 months of age. **J,** Quantification of the endomucin-positive pancreatic neoplastic areas revealed that the percentage of endomucin -positive neoplastic area in the PKC mice was significantly higher than that of the N4*^−^^/^^−^*PKC mice at the time point of 5 months of age. **, *P* < 0.01 by *t* test. Folds of magnification are 400× for A-D and 200× for **E**, **F**, **H**, **I**, respectively.

Because the expression and activation of Notch4 are known to be associated with endothelial components and vascular development (11, 29, 53–57), we investigated the expression levels of Notch4-ICD in the endothelial components in the pancreatic tissues of caerulein-treated KC GEMM (Fig. 5) using IF double labeling of Notch4-ICD and endomucin, an endothelial cell marker. Consistent with the previous literature (29, 57), we observed colocalization of Notch4-ICD and endomucin by IF (Fig. 5A–D), which was further verified by IHC (Supplementary Fig. S7).

To investigate whether loss of Notch4 expression in the endothelial compartment might have contributed to the attenuated ADM and PanIN formation *in vivo*, we first examined the endothelial cells in the KC and N4^−/−^KC mice at day 7 as well as at day 21 after the caerulein treatment using IHC of endomucin (Fig. 5E–G, Supplementary Fig. S8). At day 7 after the caerulein treatment, the endomucin expression levels were significantly higher in the KC mice group compared with the N4^−/−^KC mice group (*P* < 0.05; Fig. 5E–G). However, this difference in the endomucin expression levels between the two groups diminished by day 21 after caerulein treatment (Supplementary Fig. S8C). To assess potential long-term impact, we also examined the endothelial cells in the PKC and N4^−/−^PKC mice at the age of 2 and 5 months by IHC (Supplementary Fig. S9–10, Fig. 5H–J). The pancreases of the N4*^−^^/^^−^*PKC mice tended to display lower endomucin expression than those of the PKC mice at the age of 2 months, albeit the differences did not reach statistical significance in both the neoplastic area (*P* = 0.055; Supplementary Fig. S9A–S9C) and the normal area (*P* = 0.063; Supplementary Fig. S9D–S9F). At the age of 5 months, the endomucin expression levels were significantly higher in the PKC mice group compared with the N4^−/−^PKC mice group in both the neoplastic area (*P* < 0.01; Fig. 5H–J) and the normal area (*P* < 0.05; Supplementary Fig. S10). These results demonstrate that loss of *Notch4* was associated with a reduction in endomucin+ blood vessels consistent with reduced tumor angiogenesis. This reduction in blood vessel density was observed in the early stage of pancreatic tumorigenesis and continued throughout the progression of pancreatic tumor development.

Decreased fibrosis was also observed when comparing caerulein-treated KC versus N4^−/−^KC mice at 7 and 21 days posttreatment, and PKC versus N4^−/−^PKC groups at 2 and 5 months of age using Sirius Red staining (Supplementary Figs. S11 and S12). However, only the difference between caerulein-treated KC versus N4^−/−^KC mice at 21 days posttreatment reached statistical significance (Supplementary Fig. S11, *P* = 0.001). To assess potential changes in the inflammatory responses that may have resulted from the loss of *Notch4*, the same caerulein-treated KC versus N4^−/−^KC mice at 7 and 21 days posttreatment, and PKC versus N4^−/−^PKC groups at 2 and 5 months of age were compared using CD68 labeling. The results of these analyses indicate that there was no pronounced difference among them (Supplementary Figs. S13 and S14).

Leveraging existing scRNA-seq data from Schlesinger and colleagues, who had performed scRNA-seq experiment of pancreatic tissues taken from *Ptf1a-CreER;LSL-KrasG12D;LSL-tdTomatao* mice at different timepoints after tamoxifen injection (33), we found that in pancreatic tumorigenesis, the expression of *Notch4* is concentrated in the endothelial compartment (Supplementary Fig. S15A), with some expressions also detected in ductal cells (normal and PDAC cells, Supplementary Fig. S15B) and fibroblasts. This finding is consistent with our IHC and IF results and supports our conclusion that loss of *Notch4* induced profound impacts on the tumor and stromal compartments, resulted in attenuated pancreatic tumorigenesis. Interestingly, we also observed that each member of the *Notch* gene family has unique expression patterns in the pancreas. Different from *Notch4*, the expressions of *Notch1* and *Notch2* are more ubiquitous, whereas *Notch3* expression is majorly detected in the pericyte compartment. These data suggest that each gene member may possess some nonoverlapping functions in the pancreas.

### *Notch4* Regulates *Pcsk5* Expression in PanIN and PDAC and Decreased Expression of Pcsk5 Correlates with Good Survival

**FIGURE 6:**
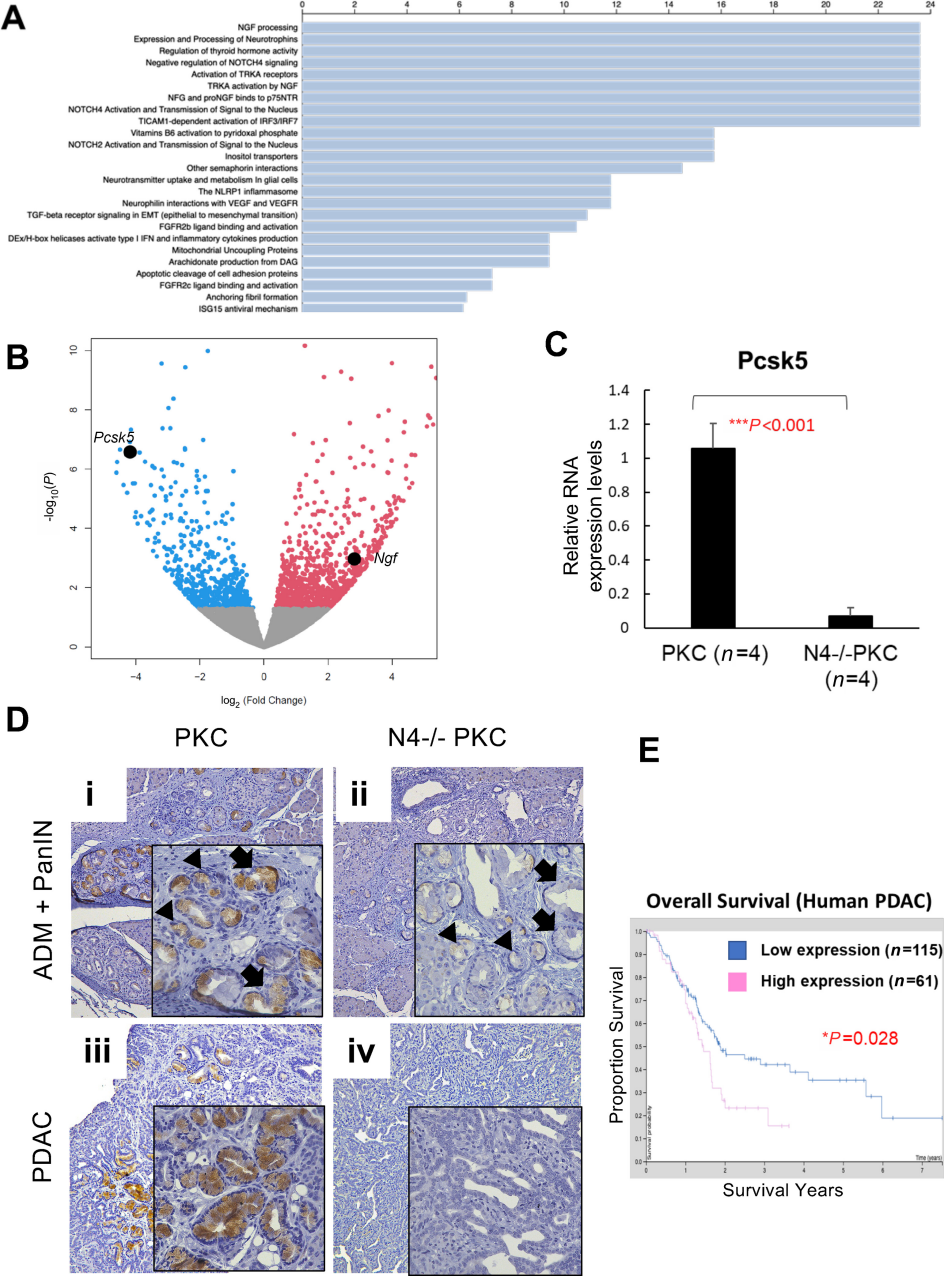
Loss of *Notch4* resulted in the downregulation of *Pcsk5* expression in pancreatic cancer. **A,** Statistically significantly differentially expressed genes of RNA-seq analyses between PKC versus N4^−/−^PKC pancreatic tumor cell lines (FDR < 0.05) were compared with the Reactome Pathway database. NGF processing was the top signaling pathway associated with *Notch4* loss, thus NGF and Pcsk5 in the pathway were implicated to be downstream mediators of Notch4. **B,** Volcanoplot depicting the differentially expressed RNAs between PKC versus N4^−/−^PKC pancreatic tumor cell lines (FDR < 0.05). RNAs were organized by log_2_ fold change (*x*-axis) and *P* value (*y*-axis). The *Pcsk5* gene was one of the top ranked genes differentially expressed, with its expression decreased by 8.36-folds, with *P* = 2.66E-07 and FDR = 5.82E-05 associated with *Notch4* loss. **C,** qRT-PCR analysis confirmed the expression levels of *Pcsk5* were downregulated in the N4^−/−^PKC pancreatic tumor cell lines (*n* = 4) compared with those of the PKC pancreatic tumor cell lines (*n* = 4). ***, *P* < 0.001 by *t* test. **D,** Representative images of Pcsk5 immunolabeling of PanIN (ⅰ, ⅱ) and PDAC (ⅲ, ⅳ) in the PKC and the N4^−/−^PKC mice. Lower expression of Pcsk5 was detected in the PanIN and PDAC of the N4^−/−^PKC mice compared with the PKC mice. Arrowheads point to ADM structures while arrow point to PanIN structures (**D** ⅰ–ⅳ; folds of magnification are 100× in the panel and 400× in the window, respectively). **E**, Kaplan–Meier survival analysis of patients with PDAC showed that patients with low (*n* = 115) PCSK5 expression had better overall survival than those with high (*n* = 61) PCSK5 expression (Human Protein Atlas). *, *P* < 0.05 by log-rank test.

To delineate potential pathways whereby the inactivation of Notch4 leads to preferable outcome in PDAC, we performed RNA-seq analysis between primary pancreatic cancer cell lines established from the pancreatic tumors developed in the PKC and the N4^−/−^PKC GEMMs. The genotypes of the cancer cell lines were confirmed to match the genotypes of the mice. The analysis of the RNA-seq data revealed that 408 genes were statistically significantly differentially expressed with a FDR cutoff at <0.05. These statistically significantly differentially expressed genes were compared with the Reactome Pathway database and the two top pathways associated with the loss of the *Notch4* gene are the nerve growth factor (NGF) processing pathway and the expression and processing of neurotrophins (Fig. 6A). In these two reactome pathways, *NGF* and *Proprotein convertase Subtilisin/Kexin type5* (*Pcsk5*) were differentially expressed on the basis of the RNA-seq analyses (Fig. 6B). With the loss of *Notch4*, *NGF* expression increased by 5.64-folds, with *P* = 0.01 and FDR = 0.04, and the expression of *Pcsk5* decreased by 8.36-folds, with *P* = 2.66E-07 and FDR = 5.82E-05. To test these two candidate genes further, their RNA expression levels were evaluated between the PKC (*n* = 4) and the N4^−/−^PKC (*n* = 4) cell lines by qRT-PCR. While the expression of *NGF* was dramatically upregulated in the N4^−/−^PKC cells compared with the PKC cells, as predicted by the RNA-seq results, the difference did not reach statistical significance (*P* = 0.204; Supplementary Fig. S16). The RNA expression level of *Pcsk5* in the N4^−/−^PKC cells was statistically significantly downregulated by 15-folds compared with that of the PKC cells (*P* < 0.01), confirming the RNA-seq results (Fig. 6C). IHC using antibody against Pcsk5 showed vast downregulation of Pcsk5 protein expression in PanIN and PDAC lesions in the N4^−/−^PKC mice as compared with their PKC counterparts (Fig. 6D). Intriguingly, interrogation of the Human Protein Atlas showed that patients with low expression levels of Pcsk5 had better prognosis than those with high Pcsk5 expressions (*P* = 0.028; Fig. 6E). Together, these results suggest that the loss of *Notch4* can lead to downregulated expression of *Pcsk5* in PanIN and PDAC lesions, and may have contributed to the reduced tumor burdens and improved survival in the N4^−/−^PKC mice.

### PCs Inhibitor Suppressed the Proliferation of PDAC Cell Lines

Pcsk5 is a member of the subtilisin-like PCs family that mediates prodomain cleavage (58–60). PCs are involved in numerous malignancies such as skin (61), colon (62), prostate (63), lung (59), and gynecologic cancer (64, 65). To investigate the role of Pcsk5 in pancreatic carcinogenesis and to evaluate its utility as a therapeutic target for pancreatic cancer therapy, we employed a commercially available and effective general PCs inhibitor, CMK (decanoyl-Arg-Val-Lys-Arg-chloromethlketone), which has been successfully used to suppress growth of certain cancer types (58, 59, 61, 66, 67) but not yet tested in PDAC.

**FIGURE 7:**
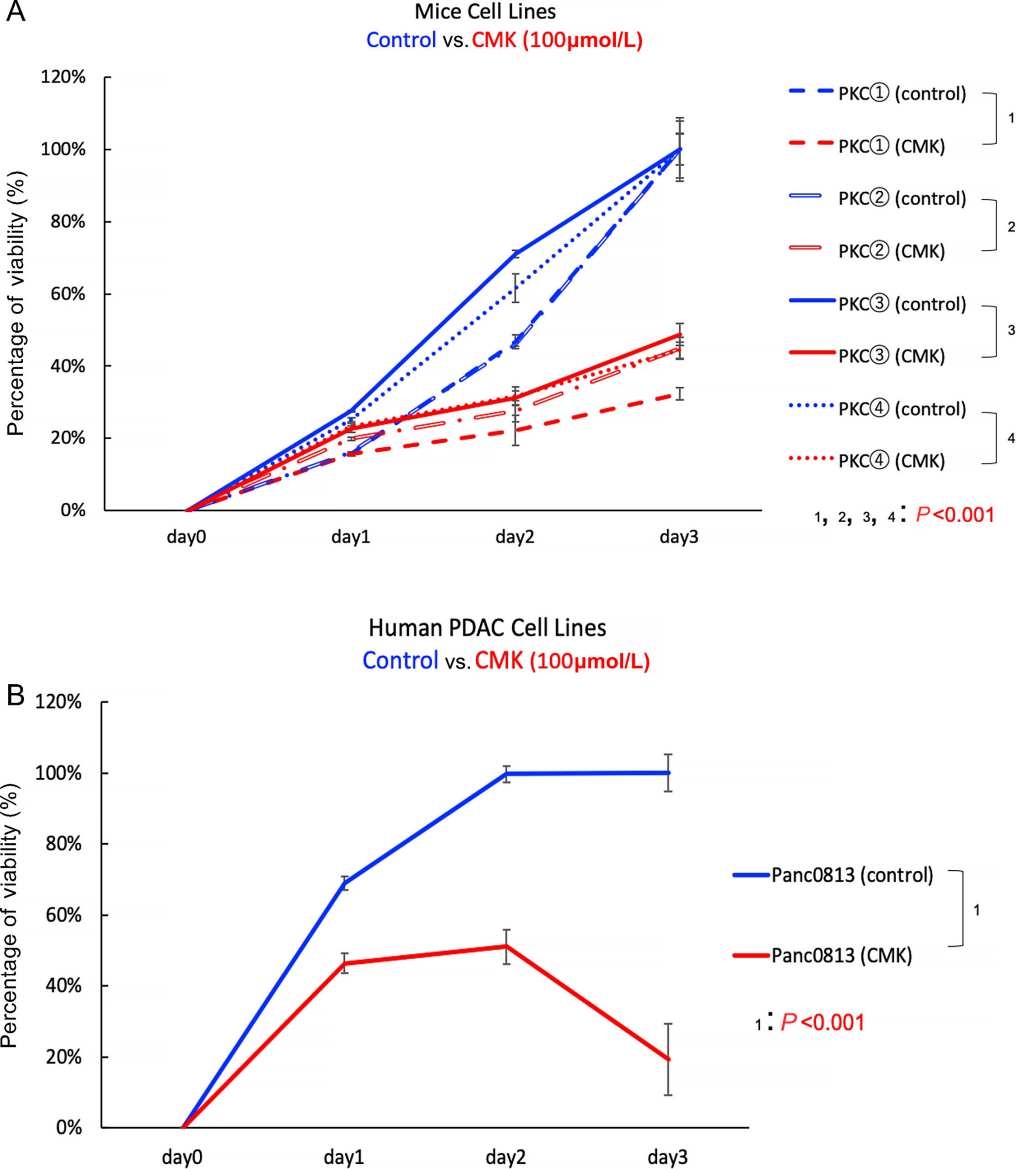
CMK suppressed the proliferation of PDAC cell lines. **A,** The proliferation of the CMK-treated (100 μmol/L) PKC cell lines were significantly lower compared with their vehicle-treated controls at day 2 and day 3 (***, *P* < 0.001). **B,** The proliferation of the CMK-treated (100 μmol/L) Panc 08.13 was significantly lower than that of the vehicle-treated control at day 1, day 2, and day 3 (***, *P* < 0.001).

Using CMK, we examined whether the inhibition of PCs is sufficient to impact PDAC growth. We compared cell proliferation between CMK-treated PKC cell lines (*n* = 4) and their vehicle-treated controls (*n* = 4) by cell proliferation assay. Cell proliferations were significantly suppressed in the CMK-treated PKC cells compared with the control group (*P* < 0.001; Fig. 7A). We also evaluated the tumor-suppressive potential of CMK using human PDAC cell line, Panc 08.13. Panc 08.13 was chosen because it has the highest *NOTCH4* RNA expression level among candidate PDAC cell lines (Supplementary Fig. S17). Similarly, we found that cell proliferation was significantly inhibited in the CMK-treated Panc 08.13 cells compared with the vehicle-treated control (*P* < 0.001; Fig. 7B). These results indicate that PCs inhibitor can suppress the growth of both murine and human PDAC cell lines, and it could be a potential novel therapeutic strategy for PDAC.

## Discussion

### Loss of *Notch4* Attenuated Both the Tumor and Stromal Components in Pancreatic Tumorigenesis

*Kras* mutations are found in over 90% of human PDAC and are thought to represent a tumor-initiating event. In addition to *Kras* mutation, inactivation of numerous tumor suppressor genes, including *P16^INK4a^*, *p53,* and *SMAD4,* increases in frequency in progressively higher PanIN stages, and culminates in PDAC (5). We have previously reported that the PKC GEMM, with the activated mutant *Kras* allele and inactivated *p16* locus, can develop a full spectrum of PanIN lesions, which progress to invasive cancer and metastasis, mimicking human pancreatic tumorigenesis at both genetic and histologic levels (22). Leveraging the high penetrance of PanIN development and PanIN to PDAC progression in the PKC GEMM (essentially 100%), we sought to investigate the potential impacts of *Notch 1* and *Notch 4* on pancreatic tumorigenesis in the current study.

Profoundly, global inactivation of *Notch4* led to improved overall survival and reduced tumor burden of the N4^−/−^PKC comparing with the PKC mice (Fig. 1). Histologic analyses of the N4^−/−^PKC mice showed that *Notch4* loss decreased ADM and PanIN formation even in early pancreatic tumorigenesis (at 2 months of age, Fig 1C). By IHC and IF, Notch4-ICD expression levels were highly upregulated in ADM and PanIN lesions compared with normal pancreas area (Fig. 2). The functional impact of *Notch4* on early pancreatic tumorigenesis was further verified using a caerulein-induced pancreatitis model, in which we found that the inactivation of *Notch4* ameliorated the structure integrity of the acinar cells and reduced the incidence of ADM *in vivo* (Fig. 3). We also confirmed that the functional impact of *Notch4* on early pancreatic tumorigenesis using explant 3D acinar cell culture experiments, in which the inactivation of *Notch4* attenuated the ADM formation *in vitro* (Fig. 4). These results indicate that global inactivation of *Notch4* can attenuate early pancreatic tumorigenesis.

Because Notch4 expression is not restricted to the tumor cells in the PKC GEMM, hence multiple cell lineages could be implicated in the observed phenotypes associated with the *Notch4* loss. We performed IHC using endomucin antibody because it has been reported that Notch4 is highly expressed in the endothelial component (11, 29, 53–57) and endomucin is considered an excellent marker for blood vessels in mouse tissues (68–70). It was developed specifically for use in mouse tissue as a mouse endothelial marker. Our stage-matched endomucin IHC analyses of the endothelial cells suggest that *Notch4* loss resulted in discernable phenotypic differences between the N4^−/−^KC and the KC GEMMs in early stage pancreatic tumorigenesis at day 7 after the caerulein treatment (Fig. 5E–G) as well as between the N4^−/−^PKC and the PKC GEMMs in the late stage of pancreatic tumorigenesis at 5 months of age (Fig. 5H–J). Reduced fibrosis was also associated with *Notch4* loss when comparing caerulein-treated KC versus N4^−/−^KC mice at 7 and 21 days posttreatment, and PKC versus N4^−/−^PKC groups at 2 and 5 months of age using Sirius Red staining (Supplementary Figs. S11 and S12). Although only the difference between caerulein-treated KC versus N4^−/−^KC mice at 21 days posttreatment reached statistical significance (Supplementary Fig. S11, *P* = 0.001), the trend of decreased fibrosis was notable in the *Notch4*-null groups. In contrast to the endothelial and fibroblast compartments, no pronounced altered inflammatory responses were detected (Supplementary Figs. S13 and S14).

Currently, we cannot yet conclude whether *Notch4* loss altered the ductal or the stromal compartment first. It is possible that the loss of *Notch4* first modified the stroma, which consequently hindered pancreatic tumor development and progression in *Notch4*-null mice. It is equally plausible that the inactivation of *Notch4* attenuated pancreatic tumorigenesis and hence reduced stromal reactivity. We postulate that Notch4 loss likely impacted both tumor and stromal compartments, as supported by the results of the scRNA-seq analysis, which revealed that Notch4 is expressed in both cellular compartments (Supplementary Fig. S15). In addition, in human PDAC, upregulation of *Notch4* transcripts have been implicated in both tumor cells and desmoplastic stroma (71). Future investigations using linage-specific Cre and conditional *Notch4* knockout GEMM to specifically target each cell type would be desirable and necessary to interrogate the role of *Notch4* in different cellular compartments comprehensively.

Intriguingly, scRNA-seq analysis also revealed unique expression patterns for each member of the *Notch* gene family. Different from *Notch4*, the expressions of *Notch1* and *Notch2* are more ubiquitous, while *Notch3* expression is majorly detected in the pericyte compartment. These data suggest that each gene member may possess some nonoverlapping functions in the pancreas. We have also previous reported that loss of Notch4 did not lead to compensatory upregulation of Notch1 expression in VEGFR3+ lymphatics (29). Hence, inhibitors that specifically target a Notch family member may serve better than pan-Notch inhibitors in inducing stronger therapeutic responses and reduced cytotoxicity, which should be taken into consideration in future therapeutic development.

### Pcsk5 May be a Novel Notch4 Downstream Mediator in the Notch4-dependent Pancreatic Tumorigenesis

Unbiased RNA-seq analyses comparing pancreatic tumor cell lines derived from the pancreatic tumors of the PKC versus the N4^−/−^PKC mice led to the identification of Pcsk5 as a potential downstream mediator of *Notch4* signaling (Fig. 6A and B). The RNA-seq result was validated by qRT-PCR results (Fig. 6C). Moreover, decreased expressions of Pcsk5 were detected in the PanIN and PDAC of the N4^−/−^PKC mice compared with those of the PKC mice (Fig. 6D). Consistent to our initial finding that the N4^−/−^PKC mice had better prognosis and overall survival than the PKC mice, the low PCSK5 expression predicts better prognosis in patients with PDAC according to the data in the Human Protein Atlas (Fig. 6E). Overall, we have demonstrated a novel association between *Notch4* expression and *Pcsk5* expression in PDAC, and the downregulation of Notch4 or Pcsk5 is associated with better prognosis in mice or human.

Pcsk5 is a member of the subtilisin-like proprotein convertase family that mediates prodomain cleavage and is one of the main acting proteins on GDF11 (growth differentiation factor 11, also known as bone morphogenetic protein, BMP11; refs. 72, 73). Inhibin α- and β-subunits, subunits that constitute inhibins and activins by heterodimerization or homodimerization, are also putative substrates of Pcsk5 (74). GDF11 and activin belong to the TGFβ superfamily, which also includes Nodal, BMPs, other growth differentiating factors, and TGFβ. Intriguingly, cross-talk between Notch4 and the activin and/or signaling axes have been reported. Sun and colleagues reported a direct interaction between the ICD of NOTCH4 and SMAD3 and demonstrated that this interaction attenuated TGFβ-mediated growth inhibition of MCF-7 breast cancer cells (75). Hardy and colleagues discovered that among the family of Notch genes, Notch4 is specifically required for the expression of Nodal in aggressive melanoma cancer cells and may be an upstream regulator of Nodal (76). Taking these previous reports into accounts, we hypothesize that *Notch4*-meidated *Pcsk5* regulation may involve cross-talk with the activin/TGFβ signaling pathways during pancreatic tumorigenesis, thus affect tumor burden and survival. Moreover, *Pcsk5* inhibition has been shown to result in inhibition of VEGF-C (77, 78), a key factor in angiogenesis and tumor nourishment and development. Hence an alternative hypothesis would be that the inactivation of *Notch4* can induce inhibition of Pcsk5 expression, resulting in impaired tumor angiogenesis and, consequently, reduced tumor burden and increased survival. The underlying mechanism regulating the interplay between *Notch4* and *Pcsk5* will require further investigations in the future.

CMK is a general PCs inhibitor (Pcsk5 included), and has been successfully used for certain cancer types (58, 59, 61, 66, 67). Current PCs research has focused on the inhibition of Furin, the prototype of the PC family. Inhibition of Furin has proved to be successful in blocking cancer cell growth and invasion in breast (79), lung (59), and head and neck cancer (80). While Furin has been reported to promote pancreatic cancer growth (81), there is no study on Pcsk5 or the efficacy of CMK in PDAC to date. In this study, we have established a potential role for Pcsk5 in PDAC and demonstrated that CMK treatment of PDAC cell lines resulted in inhibition of cell proliferation *in vitro* (Fig. 7A and B) as a proof of principles. Further preclinical evaluations of CMK and other PCs inhibitors in inhibiting Pcsk5 and PDAC growth are necessary in the future. While the treatment of CMK provides encouraging preliminary data, given that the physiologic functions of PCs are vastly different and CMK is a general PCs inhibitor, the development of an inhibitor specific for Pcsk5 would be highly desirable. Future studies using Pcsk5 knockout cell lines or mice can also provide valuable insights to its role in pancreatic tumorigenesis, its relationship to Notch4, and its feasibility as a therapeutic target.

### Conventional and Conditional Deletion of *Notch1* did not Impact the Development of PDAC in *p16^fl/fl^;LSL-Kras^G12D^;p48-Cre* Mice

Unexpectedly, we found that *Notch1* did not impact either oncogenic or tumor-suppressive influence in pancreatic tumorigenesis in the context of mutant *KRAS* and inactivation of *p16*. The results from both the N1^fl/fl^PKC and the N1^+/−^PKC GEMMs were consistent, regardless whether *Notch1* was either completely deleted in the tumor cell compartment alone or heterozygous inactivated in both the tumor and tumor microenvironment (Supplementary Figs. S1–S4). Our findings differ from previous publications, one of which touted *Notch1* as a tumor-promoting gene (17), whereas the other concluded *Notch1* is a tumor-suppressor gene (18). Although both previous reports and our study used the same *LSL-Kras^G12D^* oncomouse and targeted p48-expressing cell lineages, the two previous reports only focused on the impacts of *Notch1* on the development of premalignant lesions such as ADM and PanIN, but did not evaluate potential changes in invasive PDAC and survival curve (and no engineered p16 inactivation; refs. 17, 18) as we have done in the current study. It is possible that *Notch1* plays a differential role in the early versus late pancreatic tumorigenesis. However, because we did not observe dynamic differences in the development or progression of ADM and PanIN lesions associated with *Notch1* loss (Supplementary Figs. S2 and S4), this possibility cannot fully account for the disparity between our data and the previous reports. We have previously demonstrated that loss of *p16* dramatically accelerated the development Kras-induced ADM and PanIN, their progression to PDAC and metastasis, and significantly reduced the survival of KC mice (22). Therefore, we postulate that it is likely that the function of Notch1 is context dependent, and in the event of *p16* inactivation when oncogenic *Kras*-induced ADM and PanIN are destined to progress to PDAC, the inactivation of *Notch1* becomes ineffective in inhibiting an irreversible and inevitable outcome of tumor growth. If the latter hypothesis is true, given that *p16* is often inactivated early in PanIN-2s and PanIN-3s (82–84), our data would suggest that Notch1 is not a feasible therapeutic target in invasive PDAC. While *NOTCH1* mutations are detected in human PDAC samples, it is not yet clear when *NOTCH1* is mutated during pancreatic tumorigenesis. Hence a more complete knowledge of *NOTCH1* mutation status in sequential progression from PanIN to PDAC would be necessary to provide insights to the divergent observations in these GEMMs and to address the potential therapeutic implications raised by our findings.

In summary, we have demonstrated that the global inactivation of *Notch4* attenuated pancreatic tumorigenesis in our GEMM, which indicates that *Notch4* works as a protumorigenic factor in pancreatic tumorigenesis. Our results are consistent to previous reports that suggest Notch4 possesses tumor-promoting functions in other cancer types, including head and neck cancer, breast cancer, melanoma, and gastric cancer (85–88). Our data also give hope that a Notch4-specific inhibitor (which would induce global inhibition of Notch4 in tissues) may have therapeutic value in suppressing pancreatic cancer growth. Given that Notch4 works as oncogene and Pcsk5 may be a downstream mediator in the Notch4-dependent pancreatic tumorigenesis, inhibition of Notch4 or Pcsk5 could be a novel plausible therapeutic strategy in the future.

## Supplementary Material

Supplementary Figures S1-S17 Supplementary Figure 1. Inactivation of Notch1 had no impact on pancreatic tumorigenesis driven by oncogenic Kras in the context of p16 inactivation.
Supplementary Figure 2. Histological analyses of the pancreatic tissues of the PKC, the N1+/- PKC, and the N1fl/flPKC mice revealed no significant differences.
Supplementary Figure 3. Representative H&E from the PKC, the N1+/- PKC, and the N1fl/flPKC mice at 2 months of age (PanIN, A, C, E) and 5 months of age (PDAC, B, D, F).
Supplementary Figure 4. Comparative histological analyses of PanIN lesions in the PKC, the N1+/- PKC, and the N1fl/flPKC mice revealed no significant differences.
Supplementary Figure 5. Inactivation of Notch4 reduced the formation of high-grade PanIN, although not at a statistically significant level.
Supplementary Figure 6. Representative Notch4-ICD expression in the pancreases of PKC mice
Supplementary Figure 7. The expressions of Notch4 and Endomucin were both detected in the endothelial compartment by IHC.
Supplementary Figure 8. Slight differential Endomucin expression was detected between the KC and the N4-/- KC mice by day 21 post the caerulein treatment.
Supplementary Figure 9. Inactivation of Notch4 was associated with reduced but not significant Endomucin-positive angiogenesis at 2 months of age.
Supplementary Figure 10. Inactivation of Notch4 was associated with significant reduction of Endomucin-positive angiogenesis at 5 months of age.
Supplementary Figure 11. Inactivation of Notch4 attenuated desmoplastic reaction in the pancreases treated by Caerulein.
Supplementary Figure 12. Reduced desmoplasia was observed in N4-/- PKC mice when compared to PKC mice.
Supplementary Figure 13. Inactivation of Notch4 did not alter macrophage infiltration in the pancreases treated by Caerulein.
Supplementary Figure 14. Inactivation of Notch4 did not alter macrophage infiltration in the pancreatic tumors induced by oncogenic Kras.
Supplementary Figure 15. scRNA-Seq analysis revealed Notch4 expression in the endothelial and CK19+ normal and tumor cells in pancreatic tumorigenesis.
Supplementary Figure 16. Increased expression of the NGF gene was observed with Notch4 inactivation, but not with a statistically significant association.
Supplementary Figure 17. Notch4 RNA expression level in Panc 08.13 was the highest among candidate human PDAC cell lines.
