# Supplementary material for: Inactivation of Notch4 Attenuated Pancreatic Tumorigenesis in Mice
Source: Cancer Res Commun. 2022 Dec 12;2(12):1601–16. doi: 10.1158/2767-9764.CRC-22-0106 (PMC10035463; doi:10.1158/2767-9764.CRC-22-0106)
Supplement: Supplementary Figures S1-S17 — Supplementary Figure 1. Inactivation of Notch1 had no impact on pancreatic tumorigenesis driven by oncogenic Kras in the context of p16 inactivation. Supplementary Figure 2. Histological analyses of the pancreatic tissues of the PKC, the N1+/- PKC, and the N1fl/flPKC mice revealed no significant differences. Supplementary Figure 3. Representative H&E from the PKC, the N1+/- PKC, and the N1fl/flPKC mice at 2 months of age (PanIN, A, C, E) and 5 months of age (PDAC, B, D, F). Supplementary Figure 4. Comparative histological analyses of PanIN lesions in the PKC, the N1+/- PKC, and the N1fl/flPKC mice revealed no significant differences. Supplementary Figure 5. Inactivation of Notch4 reduced the formation of high-grade PanIN, although not at a statistically significant level. Supplementary Figure 6. Representative Notch4-ICD expression in the pancreases of PKC mice Supplementary Figure 7. The expressions of Notch4 and Endomucin were both detected in the endothelial compartment by IHC. Supplementary Figure 8. Slight differential Endomucin expression was detected between the KC and the N4-/- KC mice by day 21 post the caerulein treatment. Supplementary Figure 9. Inactivation of Notch4 was associated with reduced but not significant Endomucin-positive angiogenesis at 2 months of age. Supplementary Figure 10. Inactivation of Notch4 was associated with significant reduction of Endomucin-positive angiogenesis at 5 months of age. Supplementary Figure 11. Inactivation of Notch4 attenuated desmoplastic reaction in the pancreases treated by Caerulein. Supplementary Figure 12. Reduced desmoplasia was observed in N4-/- PKC mice when compared to PKC mice. Supplementary Figure 13. Inactivation of Notch4 did not alter macrophage infiltration in the pancreases treated by Caerulein. Supplementary Figure 14. Inactivation of Notch4 did not alter macrophage infiltration in the pancreatic tumors induced by oncogenic Kras. Supplementary Figure 15. scRNA-Seq analysis revealed Notch4 expressio [file crc-22-0106-s01.pdf]

A

PKC : *p16$^{flox / flox}$; LSL-Kras^{G12D} ;p48-Cre*

N1$^{+/-}$PKC : *Notch1 $^{+ / -}$; p16$^{flox / flox}$; LSL-Kras^{G12D} ;p48-Cre*

N1$^{fl/fl}$PKC : *Notch1 $^{flox / flox}$; p16$^{flox / flox}$; LSL-Kras^{G12D} ;p48-Cre*

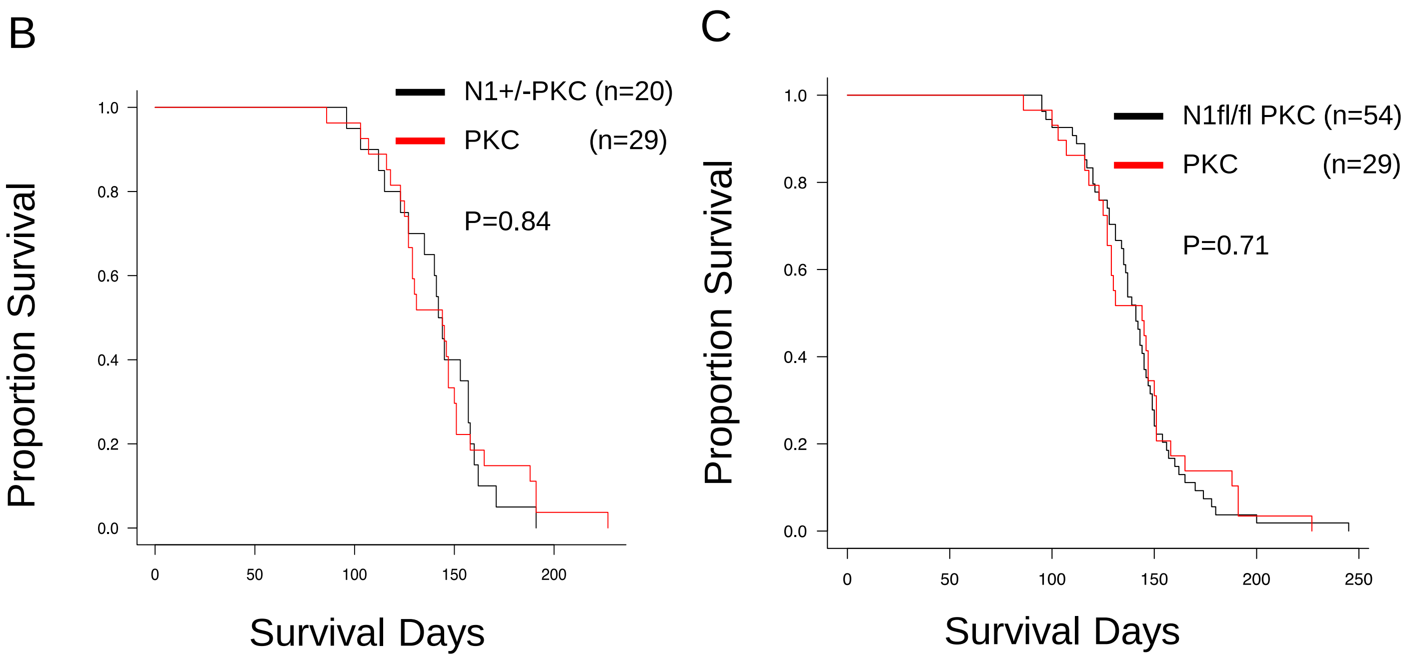

Supplementary Figure 1. Inactivation of *Notch1* had no impact on pancreatic tumorigenesis driven by oncogenic *Kras* in the context of *p16* inactivation. (A) Schematics of mouse models of pancreatic cancer, in which the PKC comprises of *p16$^{fl/fl}$* (P), *LSL-KrasG^{12D}* (K), and *p48-Cre* (C) alleles. Conventional heterozygous depletion of *Notch1* was attained by crossing the PKC with the *Notch1$^{+/-}$* mouse strain and is referred to as N1$^{+/-}$-PKC. Conditional homozygous deletion of *Notch1* was attained by crossing the PKC with the Notch1$^{fl/fl}$ mouse strain and is referred to as N1$^{fl/fl}$ PKC. (B) Kaplan-Meier analysis comparing the survival of the N1$^{+/-}$PKC (n=20) and the PKC (n=29) mice. (C) Kaplan-Meier analysis comparing the survival of the N1$^{fl/fl}$ PKC (n=54) and the PKC (n=29) mice. No statistically significant difference was observed by t-test.

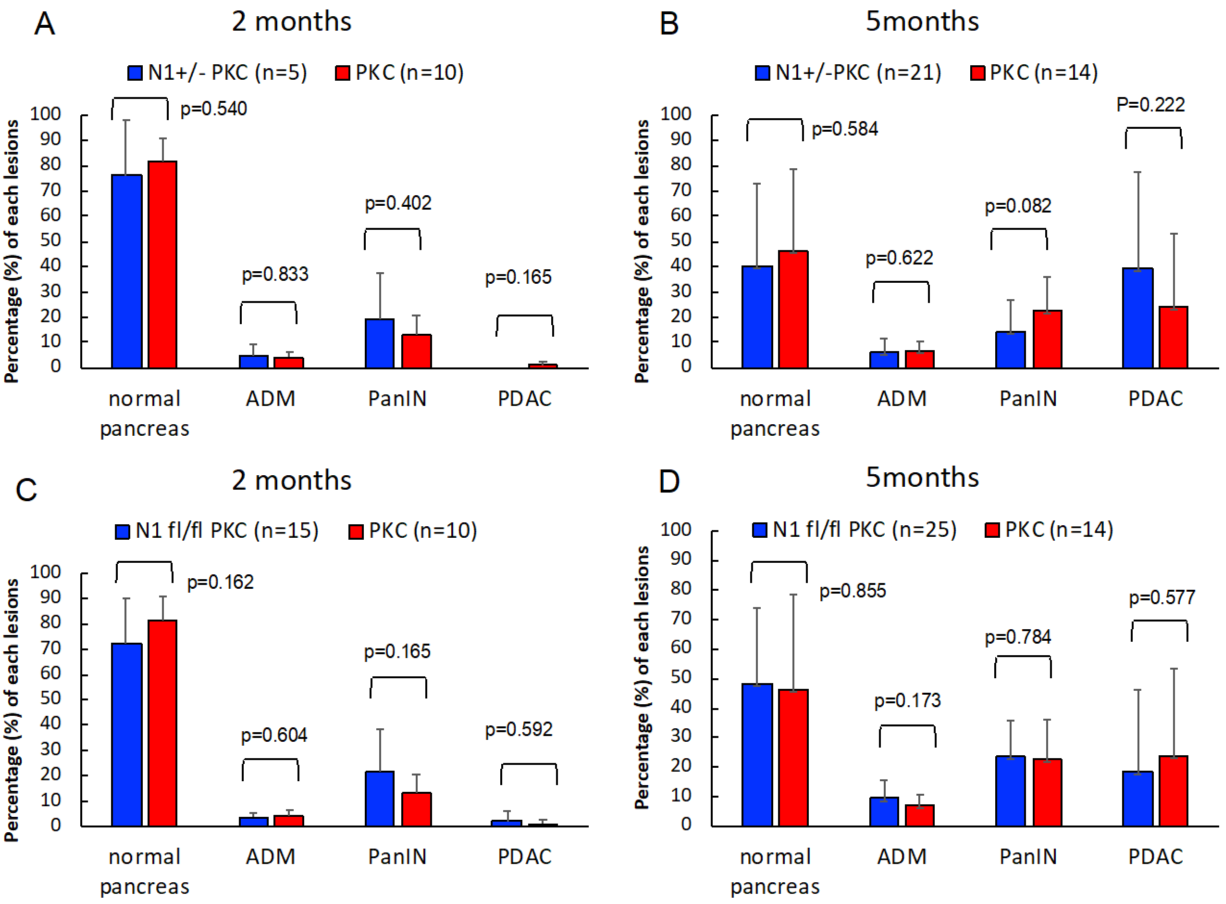

Supplementary Figure 2. Histological analyses of the pancreatic tissues of the PKC, the $N1^{+/-}$PKC, and the $N1^{fl/fl}$PKC mice revealed no significant differences.

(A, B) Evaluation of comparative percentages of the normal pancreas, ADM, PanIN, and PDAC areas between the $N1^{+/-}$PKC and the PKC mice at the ages of 2 months (A) and 5 months (B). N/S indicates not statistically significant. (C, D) Evaluation of quantitative percentages of the normal pancreas, ADM, PanIN, and PDAC areas between the $N1^{fl/fl}$ PKC and the PKC mice at the ages of 2 months (C) and 5 months (D). No statistically significant difference was observed by t-test.

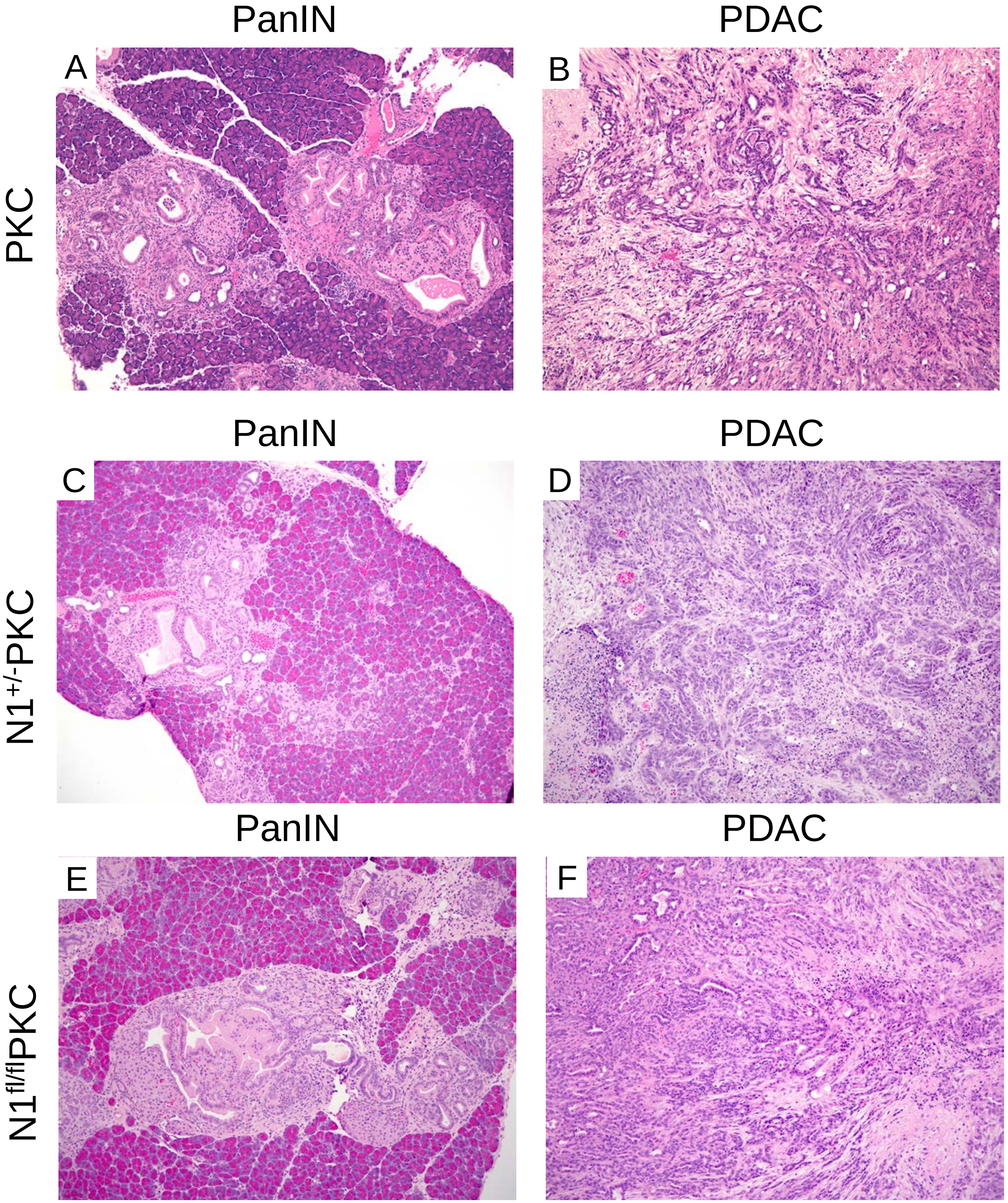

Supplementary Figure 3. Representative H&E from the PKC, the N1$^{+/-}$PKC, and the N1$^{fl/fl}$PKC mice at 2 months of age (PanIN, A, C, E) and 5 months of age (PDAC, B, D, F). Folds of magnification are x100.

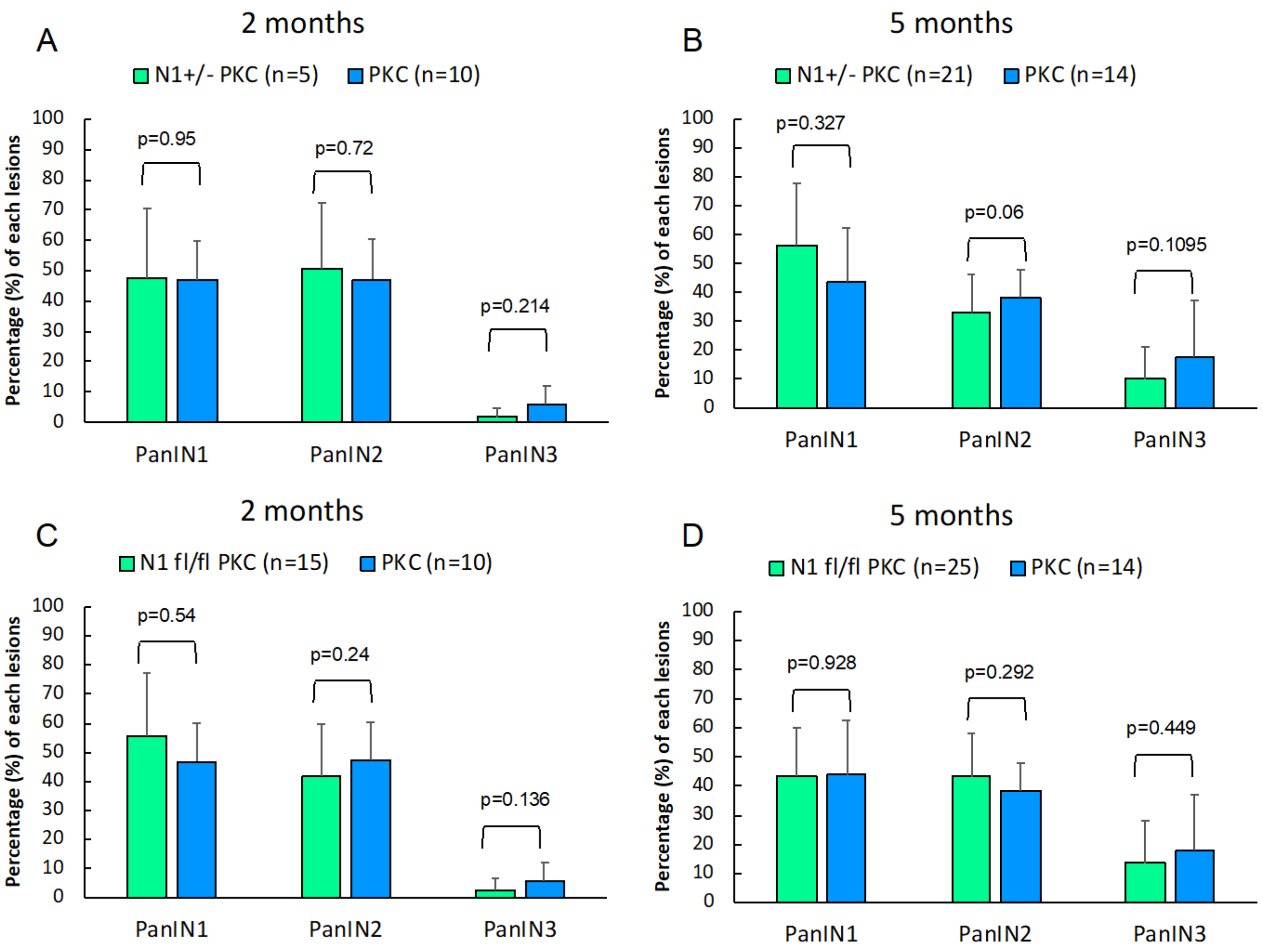

Supplementary Figure 4. Comparative histological analyses of PanIN lesions in the PKC, the N1$^{+/-}$PKC, and the N1$^{fl/fl}$PKC mice revealed no significant differences.
(A, B) Quantitative evaluation of various grades of PanIN (PanIN1, PanIN2, and PanIN3) between the N1$^{+/-}$PKC and the PKC mice at the ages of 2 months (A) and 5 months (B). N/S indicates not statistically significant. (C, D) Quantitative evaluation of various grades of PanIN (PanIN1, PanIN2, and PanIN3) between the N1$^{fl/fl}$ PKC and the PKC mice at the ages of 2 months (C) and 5 months (D). No statistically significant difference was detected by t-test.

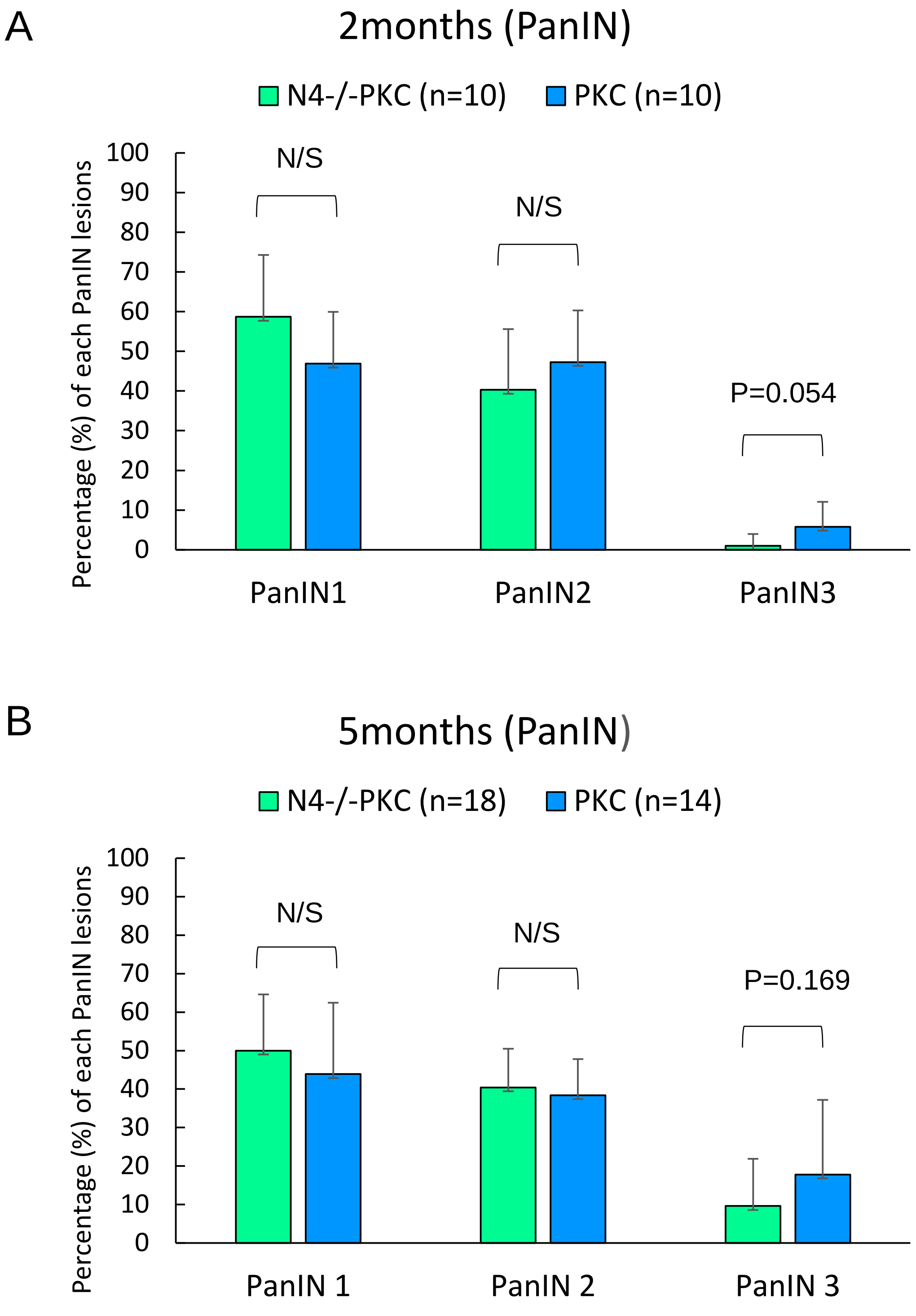

Supplementary Figure 5. Inactivation of *Notch4* reduced the formation of high-grade PanIN, although not at a statistically significant level. (A, B) Quantitative evaluation of various grades of PanIN (PanIN1, PanIN2, and PanIN3) between the N4$^{-/-}$PKC and the PKC mice at ages of 2 months (A) and 5 months (B). N/S indicates not statistically significant by t-test.

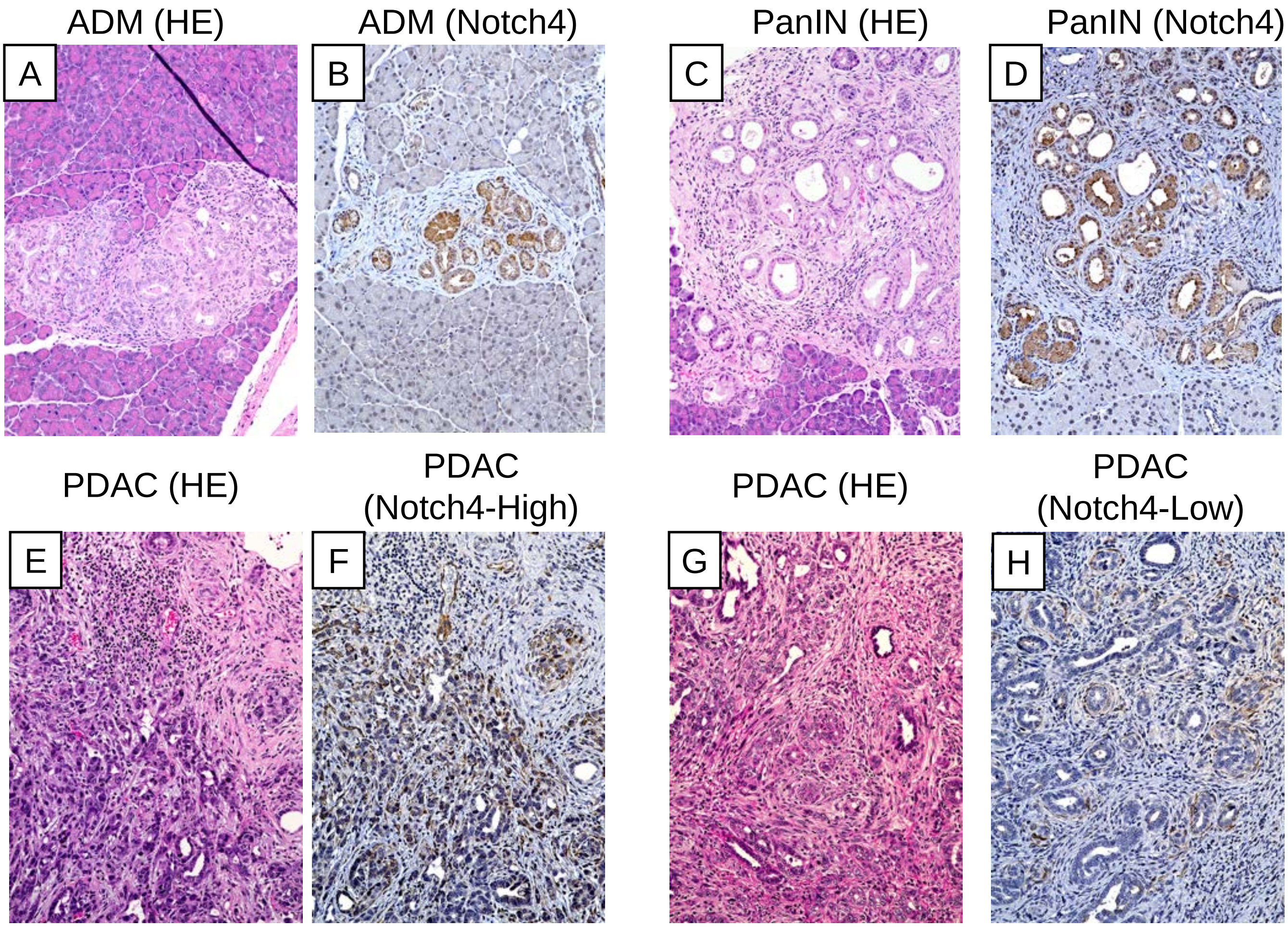

Supplementary Figure 6. Representative Notch4-ICD expression in the pancreases of PKC mice. Highly upregulated Notch4-ICD expression was detected in the ADM and PanIN lesions compared to the normal tissues by IHC (A-D). The expression level of Notch4-ICD was also elevated in PDAC regions, but the expression pattern was mixed and slightly attenuated compared to the ADM/PanIN lesions (Supplementary Fig. 6E-H). Folds of magnification are x100.

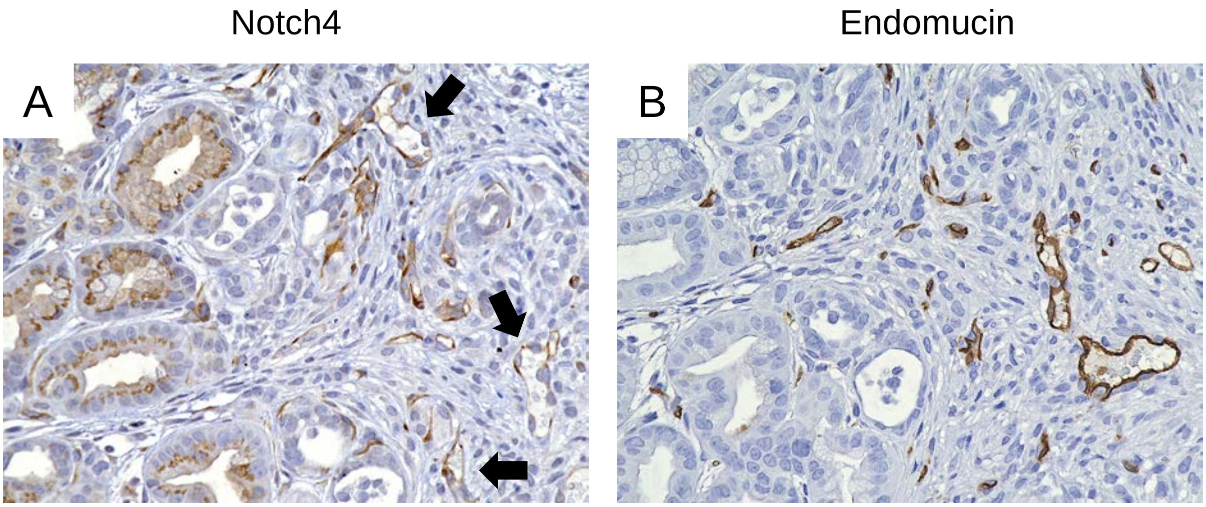

Supplementary Figure 7. The expressions of Notch4 and Endomucin were both detected in the endothelial compartment by IHC. (A, B) IHC was performed on the pancreases from the KC mice treated with Caerulein at timepoint 7 days post-treatment. Notch4 proteins were expressed not only in the ADM/PanIN lesions but also in the endothelial compartment (indicated by the arrows) (A). Endomucin proteins were detected in the endothelial compartment (B). Representative images are shown here.

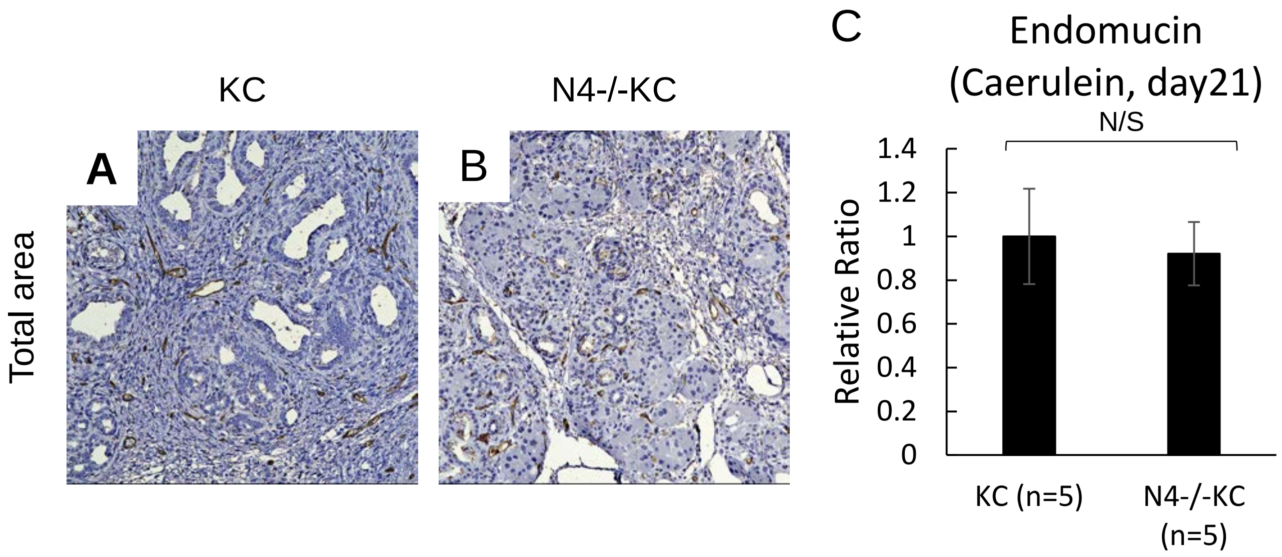

Supplementary Figure 8. Slight differential Endomucin expression was detected between the KC and the N4$^{-/-}$KC mice by day 21 post the caerulein treatment. (A, B) Endomucin IHC was performed on the pancreases from the KC (A) and the N4$^{-/-}$KC mice (B) at timepoint 21 days after the Caerulein treatment. (C) Quantification of the Endomucin-positive pancreatic areas revealed that there was no significant difference in the percentages of Endomucin -positive areas between in the KC and the N4$^{-/-}$KC mice by this timepoint. (p=0.56, not statistically significant by t-test.) N/S indicates not statistically significant by t-test.

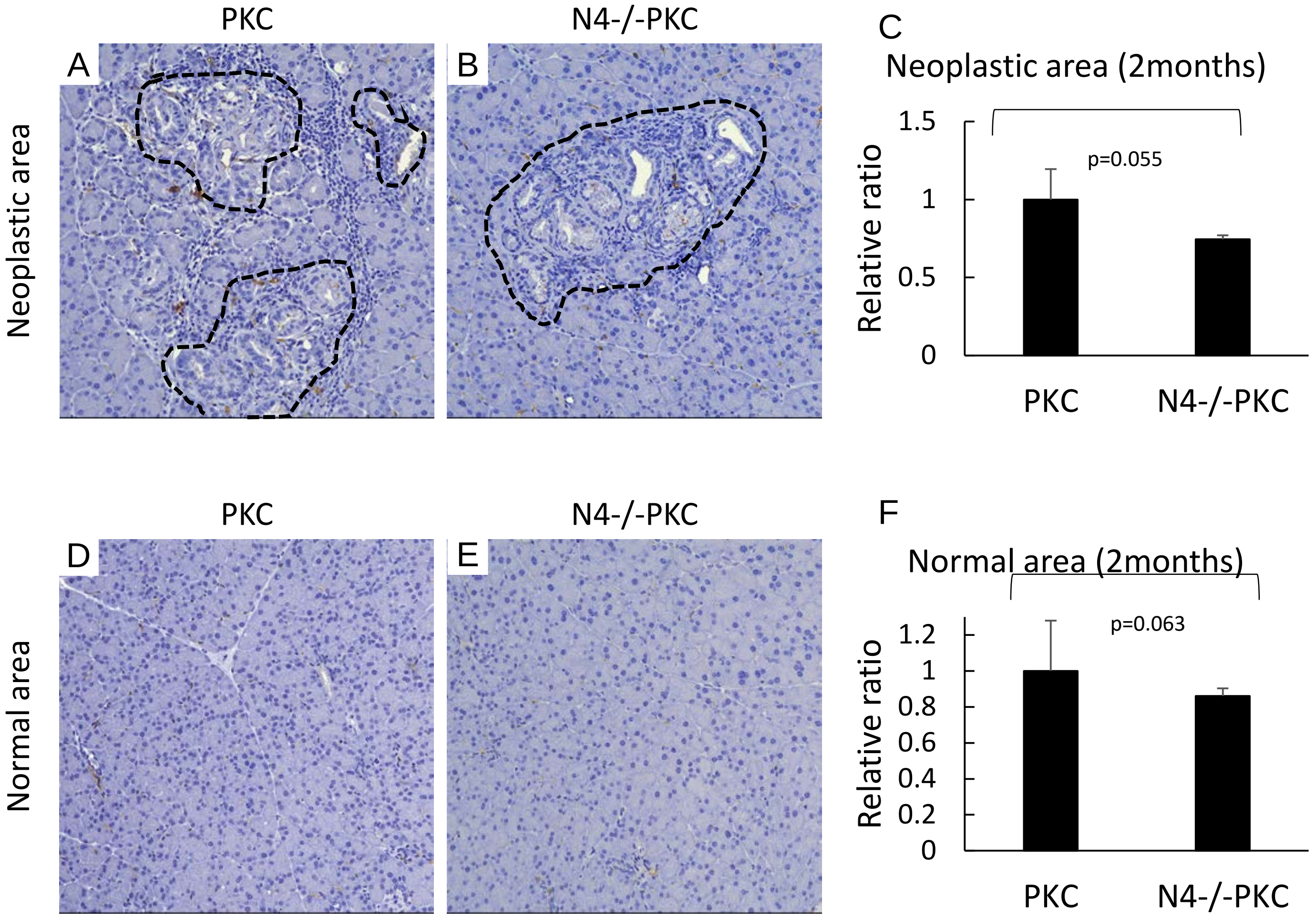

Supplementary Figure 9. Inactivation of *Notch4* was associated with reduced but not significant Endomucin-positive angiogenesis at 2 months of age. (A-F) Endomucin IHC of the neoplastic or normal areas in the pancreases of the PKC (A, D) and the N4$^{-/-}$PKC mice (B, E) at the age of 2 months. Representative IHC of the neoplastic (A, B, the areas within dotted lines) or normal areas (D, E) in theses mice. (C, F) Quantification of the Endomucin-positive pancreatic neoplastic or normal areas revealed that there was no statistically significant difference between the KC and the N4$^{-/-}$KC mice at the age of 2 months. (p=0.055 and p=0.063, respectively, by t-test.)

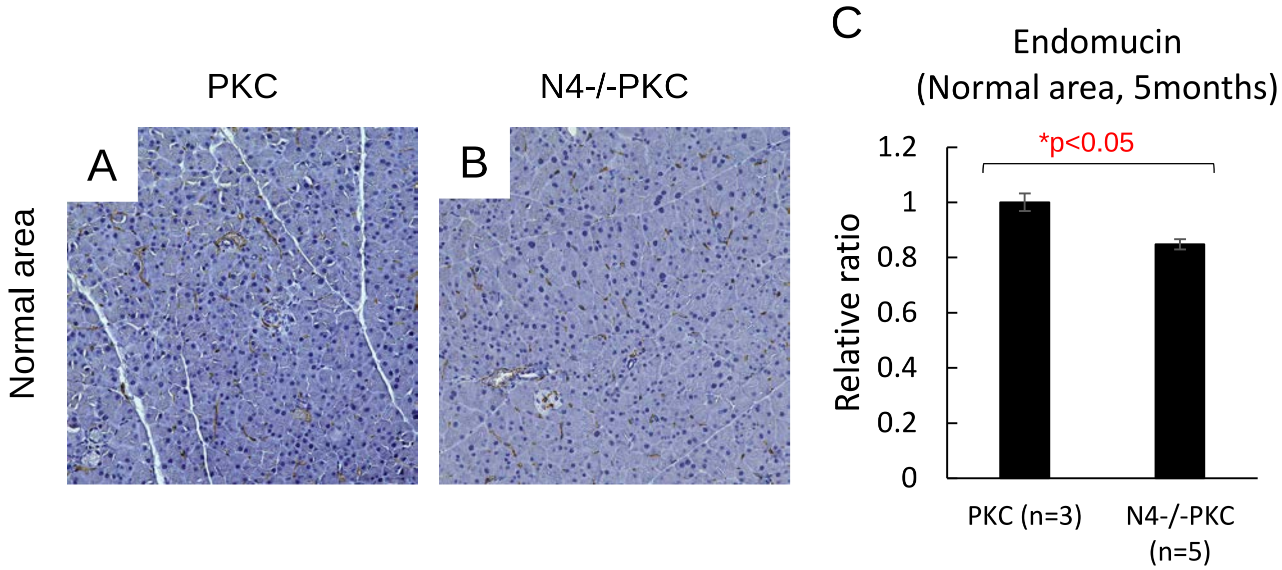

Supplementary Figure 10. Inactivation of *Notch4* was associated with significant reduction of Endomucin-positive angiogenesis at 5 months of age. (A, B) Representative Endomucin IHC of the normal area in the pancreases of the PKC (A) and the N4$^{-/-}$PKC mice (B) at the age of 5 months. Quantification of the Endomucin IHC revealed that the percentage of Endomucin -positive normal area in the PKC mice was significantly higher than that of the N4$^{-/-}$PKC mice at this timepoint. *p<0.05 by t-test.

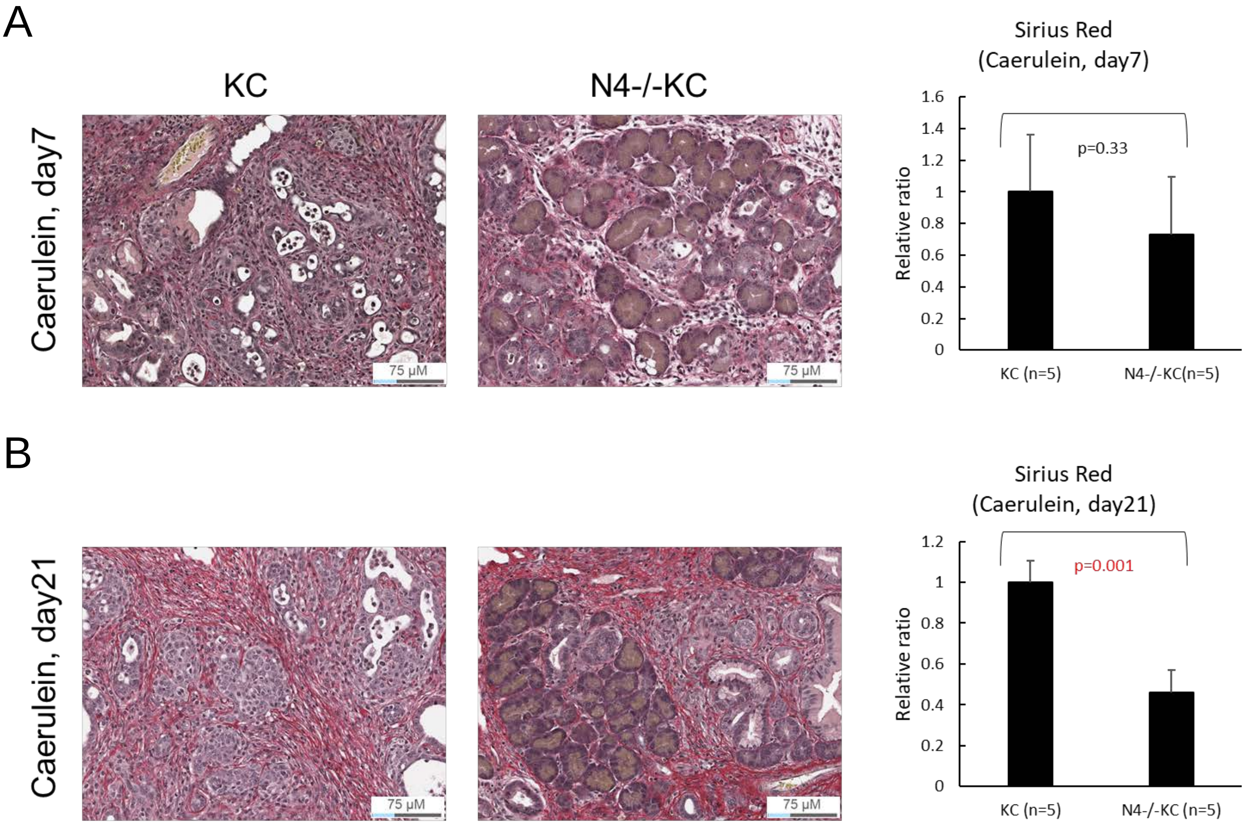

Supplementary Figure 11. Inactivation of *Notch4* attenuated desmoplastic reaction in the pancreases treated by Caerulein. Representative Sirius Red staining of the pancreases of the KC and the N4$^{-/-}$KC mice treated with the Caerulein injection at timepoints of 7 days (A) or 21 days (B) later. Folds of magnification are x200. Quantifications of the Sirius Red-positivity revealed reduced desmoplasia in the N4$^{-/-}$KC mice compared to the KC mice, but statistically significant difference was achieved only at 21 days after the caerulein treatment. p=0.001 by t-test.

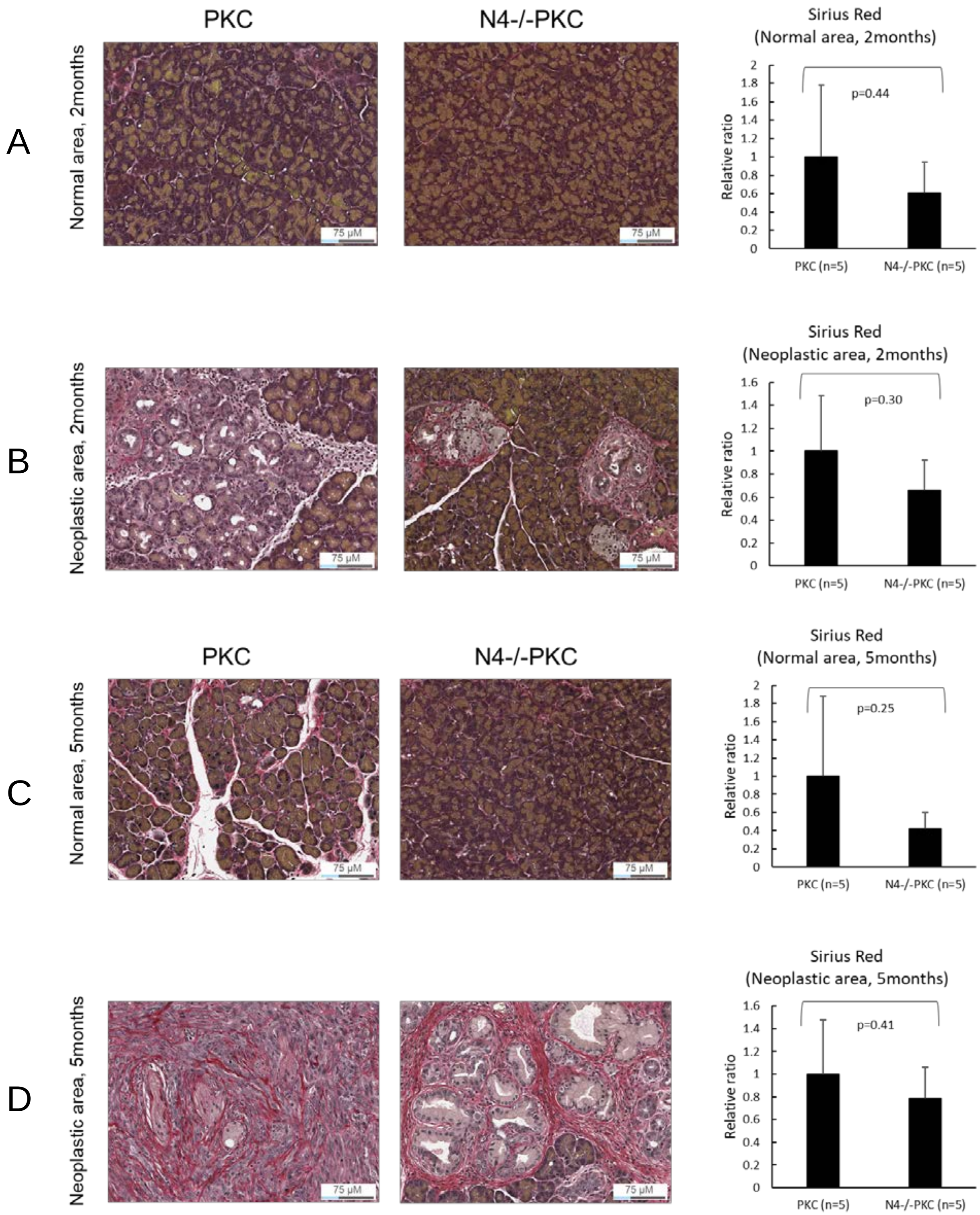

Supplementary Figure 12. Reduced desmoplasia was observed in N4$^{-/-}$PKC mice when compared to PKC mice. Representative Sirius Red staining of the pancreases of the PKC and the N4$^{-/-}$PKC mice at the age of 2 (A-B) or 5 months (C-D). Folds of magnification are x200. Quantifications of the Sirius Red-positivity revealed reduced desmoplasia in the N4$^{-/-}$PKC mice compared to the PKC mice, but without statistical significance.

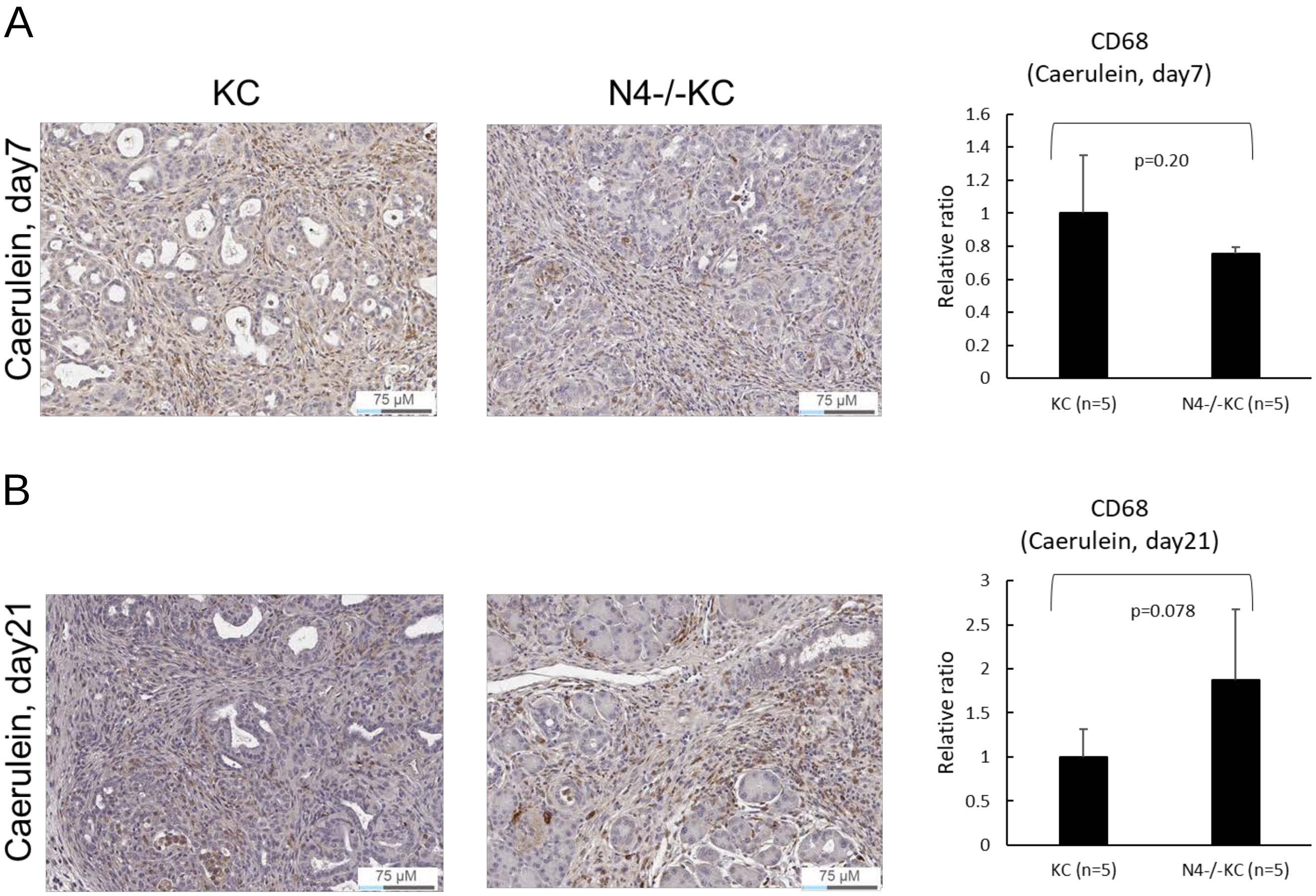

Supplementary Figure 13. Inactivation of *Notch4* did not alter macrophage infiltration in the pancreases treated by Caerulein. Representative CD68 IHC of the pancreases of the KC and the $N4^{-/-}$KC mice treated with the Caerulein injection at timepoints of 7 days (A) or 21 days (B) later. Folds of magnification are x200. Quantifications of the CD68-positivity did not detect significant differences between the two groups. Statistical significance was determined by t-test.

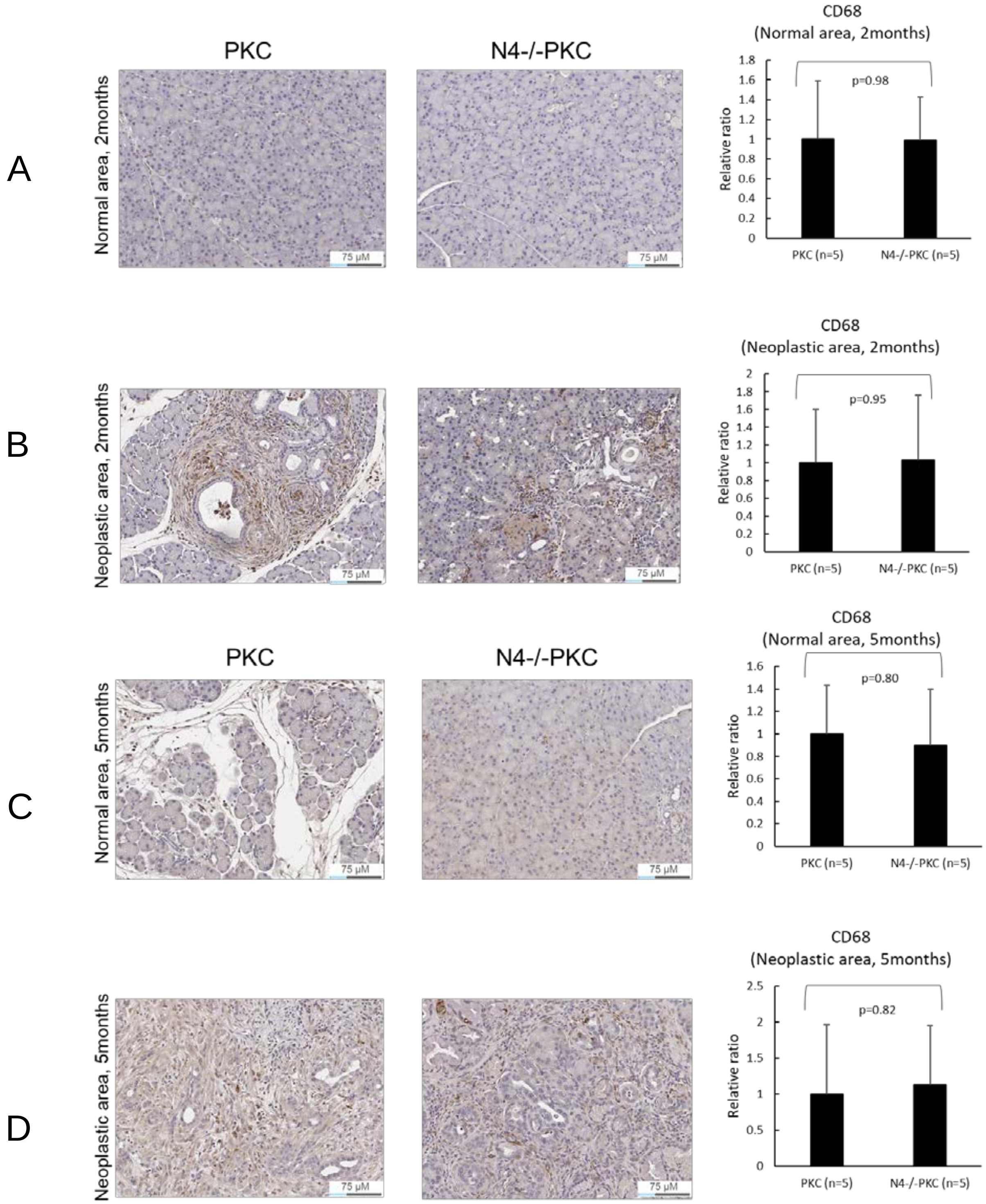

Supplementary Figure 14. Inactivation of *Notch4* did not alter macrophage infiltration in the pancreatic tumors induced by oncogenic Kras. Representative CD68 IHC of the pancreases of the PKC and the $N4^{-/-}$ PKC mice at the age of 2 (A-B) or 5 months (C-D). Folds of magnification are x200. Quantifications of the CD68-positivity did not detect significant differences between the two groups. Statistical significance was determined by t-test.

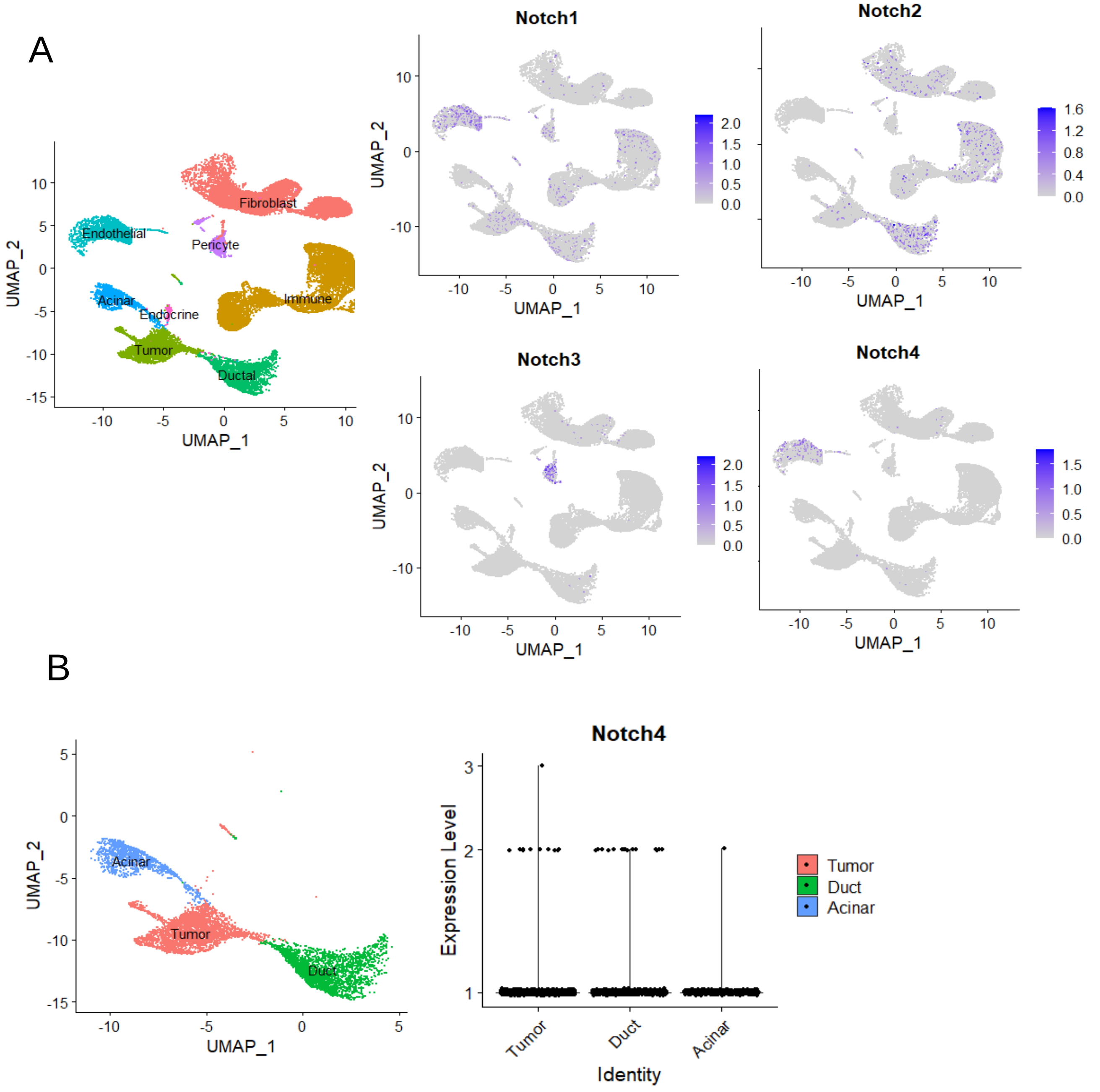

Supplementary Figure 15. scRNA-Seq analysis revealed Notch4 expression in the endothelial and CK19+ normal and tumor cells in pancreatic tumorigenesis. (A) UMAP clustering from scRNA-Seq analysis of pancreatic tissues taken from *Ptf1a-CreER;LSL-KrasG12D;LSL-tdTomatao* mice. Differential expression patterns of the *Notch* family genes (*Notch 1-4*) were detected in pancreatic tumorigenesis, with the expression of *Notch4* concentrated in the endothelial compartment, with some expression also detected in the fibroblast and ductal cells. (B) When the data is subset to only the acinar, tumor and ductal cells, violin plot showed that for *Notch 4* expressions were also detected in CK19+ PDAC and normal ductal cells.

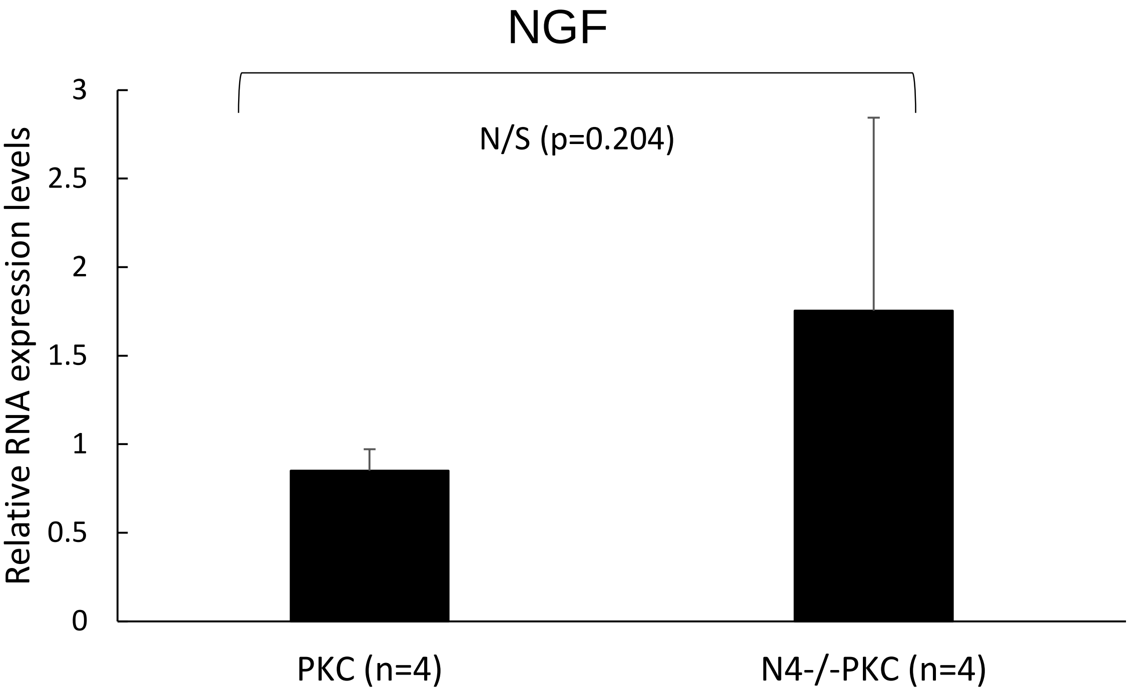

Supplementary Figure 16. Increased expression of the *NGF* gene was observed with *Notch4* inactivation, but not with a statistically significant association. (A) Quantitative RT-PCR analysis showed that there was no significant difference of the expression levels of NGF between pancreatic tumor cell lines derived from the N4$^{-/-}$PKC mice (n=4) and the PKC mice (n=4). N/S indicates not statistically significant.

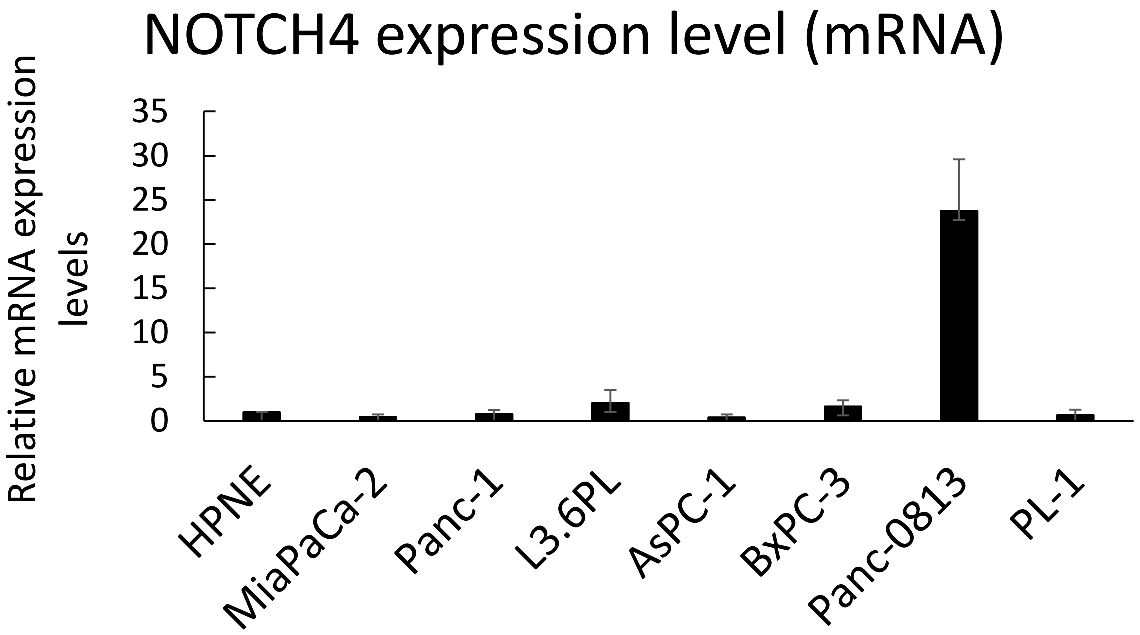

Supplementary Figure 17. Notch4 RNA expression level in Panc 08.13 was the highest among candidate human PDAC cell lines. The expression level of Notch4 in Panc 08.13 was 23.75 times higher compared to HPNE (normal pancreas cell line).
